# Overall assessment of antibiotic substitutes for pigs: a set of meta-analyses

**DOI:** 10.1186/s40104-020-00534-2

**Published:** 2021-01-07

**Authors:** Bocheng Xu, Jie Fu, Luoyi Zhu, Zhi Li, Mingliang Jin, Yizhen Wang

**Affiliations:** National Engineering Laboratory of Biological Feed Safety and Pollution Prevention and Control, Key Laboratory of Animal Nutrition and Feed of Ministry of Agriculture, Key Laboratory of Animal Nutrition and Feed Science of Zhejiang Province, Institute of Feed Science, Zhejiang University, 866 Yuhangtang Road, Hangzhou, 310058 Zhejiang Province People’s Republic of China

**Keywords:** Antibiotic substitutes, Dose-effect relationship, Feed additives, Meta-analysis, Network meta-analysis, Pigs

## Abstract

**Background:**

Antibiotic growth promoters are widely used to improve weight gain. However, the abuse of antibiotics can have many negative effects on people. Developing alternatives to antibiotics is an urgent need in livestock production. We aimed to perform a meta-analysis and network meta-analysis (NMA) to investigate the effects of feed additives as potential antibiotic substitutes (ASs) on bacteriostasis, growth performance, intestinal morphology and immunity. Furthermore, the primary, secondary, and tertiary ASs were defined by comparing their results with the results of antibiotics.

**Results:**

Among 16,309 identified studies, 37 were summarized to study the bacteriostasis effects of feed additives, and 89 were included in the meta-analysis and NMA (10,228 pigs). We summarized 268 associations of 57 interventions with 32 bacteria. The order of bacteriostasis effects was as follows: antimicrobial peptides (AMPs) ≈ antibiotics>organic acids>plant extracts>oligosaccharides. We detected associations of 11 feed additives and 11 outcomes. Compared with a basal diet, plant extract, AMPs, probiotics, microelements, organic acids, bacteriophages, lysozyme, zymin, and oligosaccharides significantly improved growth performance (*P* < 0.05); organic acids, probiotics, microelements, lysozyme, and AMPs remarkably increased the villus height:crypt depth ratio (V/C) (*P* < 0.05); and plant extracts, zymin, microelements, probiotics, and organic acids notably improved immunity (*P* < 0.05). The optimal AMP, bacteriophage, lysozyme, microelements, oligosaccharides, organic acids, plants, plant extracts, probiotics, and zymin doses were 0.100%, 0.150%, 0.012%, 0.010%, 0.050%, 0.750%, 0.20%, 0.040%, 0.180%, and 0.100%, respectively. Compared with antibiotics, all investigated feed additives exhibited no significant difference in effects on growth performance, IgG, and diarrhoea index/rate (*P* > 0.05); AMPs and microelements significantly increased V/C (*P* < 0.05); and zymin significantly improved lymphocyte levels (*P* < 0.05). Furthermore, linear weighting sum models were used to comprehensively estimate the overall impact of each feed additive on pig growth and health.

**Conclusions:**

Our findings suggest that AMPs and plant extracts can be used as primary ASs for weaned piglets and growing pigs, respectively. Bacteriophages, zymin, plants, probiotics, oligosaccharides, lysozyme, and microelements can be regarded as secondary ASs. Nucleotides and organic acids can be considered as tertiary ASs. Future studies should further assess the alternative effects of combinational feed additives.

## Background

Antibiotics are widely used in commercial pig production for growth promotion and disease prevention [1]. Subtherapeutic doses of antibiotics are used as feed additives to promote growth performance, improving average daily gain (ADG) and gain:feed ratio (G/F) through alterations in intestinal morphology and digestion and the suppression of harmful bacteria [2]. However, feeding pigs subtherapeutic doses of antibiotics in the long term leads to the development of antimicrobial resistance, which is seriously endangering public health [3]. Considering its harm, in 1999, the European Union banned the use of subtherapeutic doses of antibiotics in livestock [3]. In 2017, the FDA reported that antibiotics that are important for human medicine could no longer be used for growth promotion in food animals [4]. Notably, China will completely ban the use of antibiotics in feed in 2020 [5]. Therefore, governments and world organizations have initiated a series of countermeasures and encouraged the research and development of antibiotic substitutes (ASs). However, some questions about ASs are the following. 1) How should ASs be defined? 2) What are the effects of many feed additives on bacteriostasis, growth promotion, improvement of intestinal morphology and immunity? 3) What is the optimal dose for these feed additives? 4) Which additive is the most powerful AS? In this study, we performed a set of meta-analyses to investigate the effects of different feed additives regarded as ASs on growth performance, intestinal morphology and immunity in pigs. Then, we used network meta-analyses (NMAs) to assess and compare the effects of antibiotics and different ASs that are superior to the basal diet. Finally, we used a linear weighted model to evaluate ASs. To the best of our knowledge, this study is the first to comprehensively and systematically define ASs and investigate their effects.

## Methods

This meta-analysis is reported according to the Preferred Reporting Items for Systematic Reviews and Meta-Analyses (PRISMA) Statement [6] and the Approach of Meta-analysis on Nonruminants [7, 8].

### Search strategy

We performed a series of meta-analyses of studies on potential ASs indexed on PubMed from January 1, 2000 to April 31, 2019, and the language was restricted to English. The complete search strategy is shown in Table S1. Moreover, studies on antimicrobial peptides (AMPs) were identified by searches in the Antimicrobial Peptide Database (http://aps.unmc.edu/AP/, accessed on April 31, 2019). In addition, a manual search was performed to obtain additional potential studies.

### Selection criteria

The inclusion criteria were as follows: 1) studies investigating the effects of ASs on bacteriostasis; 2) studies investigating the effects of potential ASs as feed additives on pig growth performance, the villus height:crypt depth ratio, blood haematology, or diarrhoea; and 3) studies in which the breeding background was commercial pigs. The exclusion criteria were as follows: 1) studies on antibacterial effects that did not report minimum inhibitory concentrations (MICs) or animal-specific bacteria; 2) studies without a basal diet or a positive control group; 3) studies where pig growth was not assessed in stages; 4) studies in which pigs were challenged with pathogenic bacteria, viruses, or lipopolysaccharide; 5) studies that included multiple factors; and 6) studies in which pigs exhibited an oxidative stress status or a heat stress state.

Three investigators (B. Xu, L. Zhu and J. Fu) reviewed study titles, abstracts, and full texts to ensure studies satisfied the inclusion criteria, and disagreements were resolved by two investigators (M. Jin and Y. Wang).

### Information extraction

The following data were extracted from each selected study: author information (first author, year, country), interventions, control group, breeding background, amount of additive, growth stages (weaned piglets, growing pigs, finishing pigs), sample size, initial and final body weight, experimental duration, and outcome data and corresponding errors, such as standard deviations or standard errors. The initial body weight of weaned piglets was lower than 15 kg, the initial body weight of growing pigs was more than 15 kg, and the initial body weight of finishing pigs was more than 45 kg. Outcomes were as follows: MIC; ADG; average daily feed intake (ADFI); G/F; V/C of duodenum, jejunum, and ileum; immune globulin (Ig), including IgA, IgM, and IgG; lymphocyte levels, diarrhoea rate, and diarrhoea index. For studies involving multiple interventions, we extracted data from all relevant interventions. For studies involving multiple concentrations, we extracted all the experimental groups with an addition amount less than 1%. When extractions from different plant tissues were used, we chose leaf extractions.

### Study quality assessment

We conducted a study quality assessment on non-ruminants (SQANR) to assess the quality of existing studies [7]. The potential risk of bias was derived from missing within-group error, repeated reports, information completeness, sample size, and experimental rationality. Two investigators (B. Xu and L. Zhu) performed independent study quality assessments.

### Statistical analysis

We aimed to compare which ASs were most suitable in terms of bacteriostasis, growth promotion and disease resistance effects. First, we compared the effects of basal diet with those of feed additive supplementation on a range of outcomes. We used a random-effects model to compute the pooled estimate of standardized mean difference (SMD) with the 95% confidence interval (CI). If the 95% CI contained a zero value, that result indicated that there was no difference. The heterogeneity was assessed with the *I*^2^ statistic [9] and Cochran’s *Q* test [10]; *I*^2^ > 50% and *P*_heterogeneity_ < 0.1 was regarded as a substantial heterogeneity.

We used sensitivity analyses to remove individual data values with large deviations from the overall level. If 10 or more trials were available, we conducted subgroup analyses and meta-regression to explore potential sources of heterogeneity. Publication bias was evaluated using Egger’s tests, for which the significance level was defined at *P* < 0.1 [11]. Second, if the data were sufficient, we used the dose-effect model to find the optimal amount of added feed additives. When the same effect size occurred at different concentrations, we chose the lowest concentration to reduce the cost. Third, we performed an NMA to further study feed additives that, when compared with basal diet, had a significant effect on growth performance. We aimed to compare the growth performance effects of feed additives with the optimal amount of ASs added. NMA enables the incorporation of indirect comparisons constructed from two trials with the same control group. NMA combined all available comparisons among ASs and provided a ranking of suitable alternatives to antibiotics [12]. To explore evidence of within-network inconsistency, the loop-specific approach was used [13].

We used Stata 14.0 (Stata Corp., USA) to perform the meta-analysis. We used R 3.6.1 (The R Foundation Conference Committee, USA) to examine the dose-effect relationship and perform the NMA.

### Assessment of antibiotic substitutes

We aimed to use a linear weighting sum model to comprehensively assess the efficacy of feed additives. In terms of bacteriostasis, according to the order of occurrences, 4 bacteria were chosen from gram-positive/negative bacteria for analysis. We used the rank score of the interventions to assess their bacteriostasis effects based on MICs. We used the *P*-score value, which evaluates and ranks the strength of the intervention from the NMA, to grade available interventions. Each *P*-score value of feed additives was subtracted by that of the corresponding basal diet, which was performed to guarantee a consistent background. When feed additives were not included in the NMA or were not observed in the outcomes, the feed additives were rated zero in the corresponding outcomes. For growth performance, the weights of ADG, ADFI, and G/F were 30%, 10%, and 60%, respectively. For intestinal morphology, the weights of V/C of the duodenum, jejunum, and ileum were 30%, 10%, and 60%, respectively. For immunity, the weights of IgA, IgM, IgG, and lymphocyte levels and diarrhoea index/rate were 10%, 10%, 10%, 10%, 60%, respectively. The overall score was equal to the sum of the score of bacteriostasis, growth performance, intestinal morphology, and immunity effects multiplied by the corresponding weight (bacteriostasis = 10%; growth performance = 50%; intestinal morphology = 10%; immunity = 30%). Furthermore, we also conducted the stage scores based on the growth stage to provide a special strategy. Feed additives that were superior to antibiotics on the stage scores were regarded as primary ASs. Feed additives that were superior to antibiotics on one outcome were regarded as secondary ASs. Finally, the remaining feed additives were regarded as tertiary ASs.

## Results

We identified 16,309 articles in PubMed, of which 89 were included in the meta-analyses [14–102], including 10,228 pigs, and 37 were summarized to investigate the antibacterial effects of feed additives [103–139]. The characteristics of the studies are shown in Table S2. The study quality assessed by SQANR is shown in Table S3. The number of studies rated as “high” and “moderate” were 7 and 61, respectively. The mean initial body weights of weaned piglets, growing pigs, and finishing pigs were 7.7 kg, 28.4 kg, and 57.6 kg, respectively. Feed additives included plant extracts, plants, probiotics, microelements, organic acids, bacteriophages, lysozyme, zymin, AMPs, nucleotides, and oligosaccharides. The results of the meta-regression are shown in Table 1. The different growth stages had a significant influence on ADFI and G/F (*P* < 0.05), while the type of feed additives and dose did not have a significant effect on the outcomes of interest (*P* > 0.05). Therefore, when we performed meta-analyses for growth performance, the growth stages were divided into weaned piglets, growing pigs, and finishing pigs.

**Table 1:** Regression analyses of the covariates

Outcomes	Type of feed additive	Growth stage	Dose
Average daily gain	0.986	0.064	0.954
Average daily feed intake	0.105	0.001	0.466
Gain: feed ratio	0.414	0.009	0.082
Villus height: crypt depth of the duodenum	0.474	0.974	0.949
Villus height: crypt depth of the jejunum	0.168	0.514	0.409
Villus height: crypt depth of the ileum	0.021	0.989	0.397
IgA	0.278	0.196	0.597
IgM	0.552	0.591	0.886
IgG	0.354	0.449	0.976
Lymphocytes	0.156	0.661	0.978
Diarrhoea rate	0.587	NA	NA
Diarrhoea index	0.535	NA	0.053

### Effects of feed additives on bacteriostasis

We summarized 268 associations of 57 interventions with 32 bacteria (Table S4). Due to the number of associations, *Staphylococcus aureus* and *Bacillus subtilis* were used to represent gram-positive bacteria, and *Escherichia coli* and *Pseudomonas aeruginosa* were used to represent gram-negative bacteria. Overall, based on the rank score, bacteriostasis effects of interventions were as follows (Table 2): AMPs≈ASs> organic acids> plant extracts> oligosaccharides, which were in accordance with bacteriostasis effects of those interventions on gram-positive or gram-negative bacteria.

**Table 2:** The rank of bacteriostasis effects

Interventions	***Staphylococcus aureus***	***Bacillus subtilis***	***Escherichia coli***	***Pseudomonas aeruginosa***
Antimicrobial peptides	1	2	1	2
Antibiotics	2	1	2	1
Organic acids	3	2	3	3
Plant extracts	4	4	5	4
Oligosaccharides	5	5	4	NA

### Effect of feed additives on growth performance

**Fig. 1:**
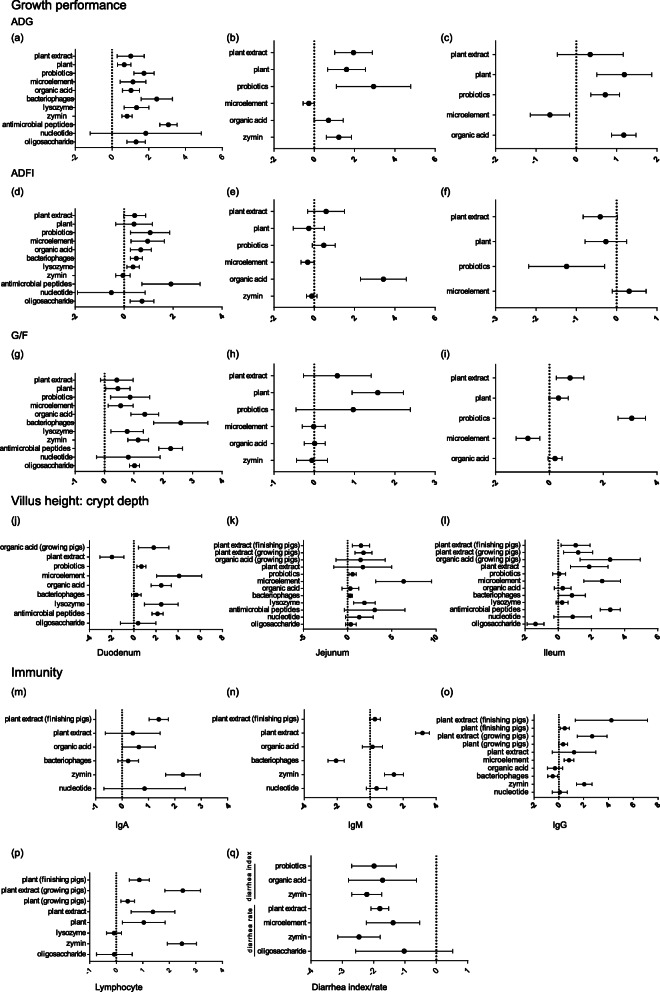
Summary forest plots of the effects of feed additives. **a** Feed additives and weaned piglets’ average daily gain. **b** Feed additives and growing pigs’ average daily gain. **c** Feed additives and finishing pigs’ average daily gain. **d** Feed additives and weaned piglets’ average daily feed intake. **e** Feed additives and growing pigs’ average daily feed intake. **f** Feed additives and finishing pigs’ average daily feed intake. **g** Feed additives and weaned piglets’ gain:feed ratio. **h** Feed additives and growing pigs’ gain:feed ratio. **i** Feed additives and finishing pigs’ gain:feed ratio. **j** Feed additives and villus height:crypt depth ratio of the duodenum. **k** Feed additives and villus height:crypt depth ratio of the jejunum. **i** Feed additives and villus height:crypt depth ratio of the ileum. **m** Feed additives and IgA level. **n** Feed additives and IgM level. **o** Feed additives and IgG level. **p** Feed additives and lymphocytes. **q** Feed additives and diarrhoea index/rate

As shown in Fig. 1a-i and Table S5, compared with a basal diet, plants and probiotics had significant effects on ADG at all stages (*P* < 0.05), plant extracts and zymin improved weaned and growing pigs’ ADG (*P* < 0.05), and bacteriophages, lysozyme, and AMPs had significant effects on weaned piglets’ ADG (*P* < 0.05), while only microelements and organic acids had no significant effect on growing pigs’ ADG (*P* > 0.05). With regard to ADFI, probiotics, microelements, organic acids, bacteriophages, lysozyme, AMPs, and oligosaccharides had a notable effect on weaned piglets (*P* < 0.05), while we detected that organic acids improved ADFI in growing pigs (*P* < 0.05) and that probiotics had a negative impact on finishing pigs’ ADFI (*P* < 0.05). In terms of G/F, plants remarkably improved weaned and growing pigs’ G/F (*P* < 0.05), probiotics had a significant effect on weaned and finishing pigs’ G/F (*P* < 0.05), and microelements, organic acids, bacteriophages, lysozyme, zymin, AMPs, and oligosaccharides had considerable effects on weaned piglets’ G/F (*P* < 0.05).

**Fig. 2:**
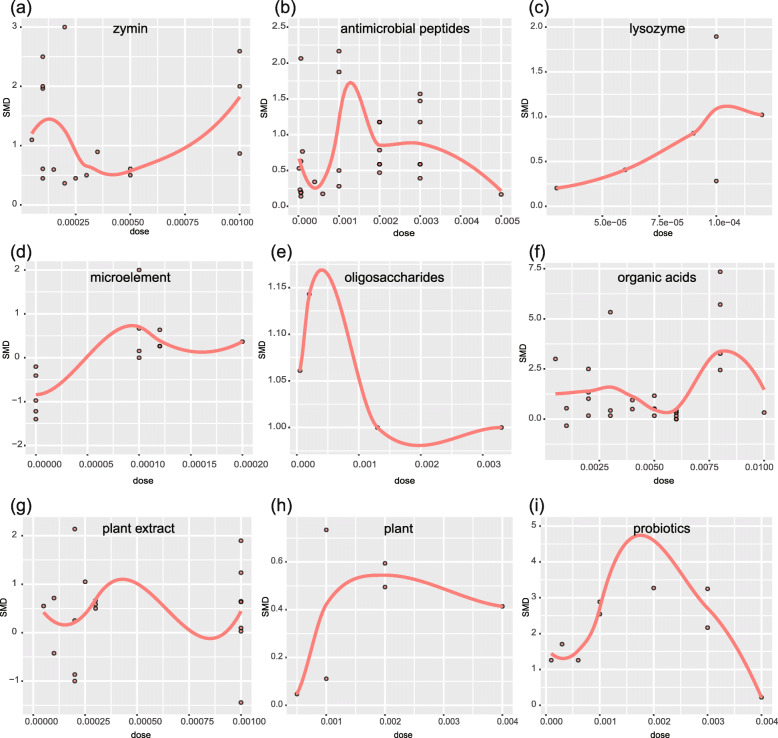
Dose-effect relationship between feed additives and growth performance. **a** Zymin. **b** Antimicrobial peptides. **c** Lysozyme. **d** Microelement. **e** Oligosaccharides. **f** Organic acids. **g** Plant. **h** Plant extract. **i** Probiotics

Figure 2 show the dose-effect relationship among the feed additives and growth performance. The optimal doses of AMPs, bacteriophages, lysozyme, microelements, oligosaccharides, organic acids, plants, plant extracts, probiotics, and zymin were 0.100%, 0.150%, 0.012%, 0.010%, 0.050%, 0.750%, 0.20%, 0.040%, 0.180%, and 0.100%, respectively.

**Fig. 3:**
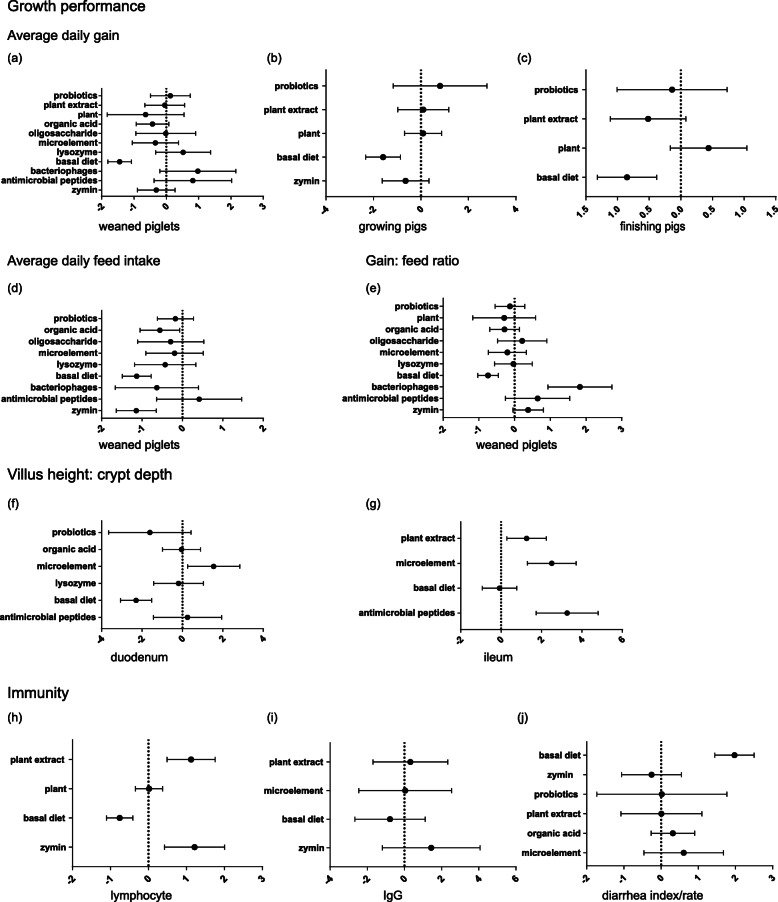
Forest plots of network meta-analysis. **a** Feed additives and weaned piglets’ average daily gain. **b** Feed additives and growing pigs’ average daily gain. **c** Feed additives and finishing pigs’ average daily gain. **d** Feed additives and weaned piglets’ average daily feed intake. **e** Feed additives and weaned piglets’ gain:feed ratio. **f** Feed additives and villus height:crypt depth ratio of the duodenum. **g** Feed additives and villus height:crypt depth ratio of the ileum. **h** Feed additives and lymphocytes. **i** Feed additives and IgG level. **j** Feed additives and diarrhoea index/rate

Figure 3 and Table 3 show the comparison between antibiotics and feed additives based on NMA. Compared with those of antibiotics, feed additives that had a positive significant effect on growth performance compared with the basal diet had no difference in growth performance. For weaned piglets’ ADG, the *P*-score values of bacteriophages, AMPs, lysozyme, and probiotics were greater than those of antibiotics. For weaned piglets’ ADFI, the *P*-score value of AMPs was greater than that of antibiotics. For weaned piglets’ G/F, the *P*-score values of AMPs, zymin, bacteriophages, and oligosaccharides were greater than those of antibiotics. For growing pigs’ ADG, the *P*-score values of probiotics, plants, and plant extracts were greater than those of antibiotics. For finishing pigs’ ADG, the *P*-score value of plants was greater than that of antibiotics. However, we did not observe a *P*-score value of feed additives greater than that of antibiotics for ADFI and G/F of growing and finishing pigs.

**Table 3:** *P*-score value table

Interventions	ADG-w	ADG-g	ADG-f	G/F-w	ADFI-w	V/C (duodenum)	V/C (ileum)	Lymphocytes	IgG	Diarrhoea rate/index
Antibiotics	0.552	0.589	0.628	0.493	0.723	0.542	0.037	0.366	0.337	0.664
Antimicrobial peptides	0.852	0.000	0.000	0.796	0.820	0.615	0.841	0.000	0.000	0.000
Bacteriophages	0.898	0.000	0.000	0.980	0.299	0.000	0.000	0.000	0.000	0.000
Basal diet	0.000	0.000	0.000	0.000	0.000	0.000	0.000	0.000	0.000	0.000
Lysozyme	0.790	0.000	0.000	0.454	0.412	0.467	0.000	0.000	0.000	0.000
Microelements	0.331	0.000	0.000	0.319	0.568	0.937	0.686	0.000	0.370	0.327
Oligosaccharides	0.524	0.000	0.000	0.622	0.500	0.000	0.000	0.000	0.000	0.000
Organic acids	0.256	0.000	0.000	0.244	0.311	0.519	0.000	0.000	0.000	0.433
Plants	0.235	0.647	0.914	0.286	0.000	0.000	0.000	0.385	0.000	0.000
Plant extracts	0.515	0.646	0.252	0.000	0.000	0.000	0.399	0.856	0.496	0.735
Probiotics	0.625	0.843	0.531	0.371	0.587	0.139	0.000	0.000	0.000	0.000
Zymin	0.334	0.266	0.000	0.755	0.001	0.000	0.000	0.893	0.740	0.807

*w* Weaned piglets, *g* Growing pigs, *f* Finishing pigs

### Effect of feed additives on intestinal morphology

As shown in Fig. 1j-l and Table S5, probiotics, organic acids, microelements, lysozyme, AMPs, plant extracts significantly improved the V/C of duodenum and ileum in weaned piglets (*P* < 0.05), while plant extracts notably improved the V/C of jejunum and ileum in growing and finishing pigs (*P* < 0.05).

As shown in Fig. 3f-g and Table 3, in weaned piglets, microelements were greater than AS on V/C of the duodenum and ileum, plant extracts and AMPs were greater than AS on V/C of the ileum, and probiotics, organic acids, lysozyme, and AMPs had no difference with AS on V/C of the duodenum. Specifically, the *P*-score value of microelements and AMPs was greater than that of AS on V/C of ileum.

### Effect of feed additives on immunity

As shown in Fig. 1m-q and Table S5, plant extracts, zymin, and microelements were associated with improved Ig levels (*P* < 0.05); plants, plant extracts, and zymin were associated with improved lymphocyte levels (*P* < 0.05); and probiotics, organic acids, zymin, plant extracts, and microelements were associated with reduced diarrhoea index/rate (*P* < 0.05).

Figure 3h-j and Table 3 show that compared with ASs, zymin significantly improved lymphocyte levels. In terms of *P*-score value, plant extracts and zymin were better than ASs at reducing and alleviating diarrhoea; plant extracts, zymin, and plants were better than ASs at increasing lymphocyte levels; and plant extracts, zymin, and microelements were better than ASs at increasing IgG levels.

### Feed additives assessment

Table 4 shows the assessment score of feed additives. The findings suggest that AMPs could be regarded as primary ASs in weaned piglets and that plant extracts could be considered ASs in growing pigs. Secondary ASs included bacteriophages, zymin, plants, probiotics, oligosaccharides, lysozyme, and microelements. In terms of bacteriostasis, AMPs were observed to have an antibacterial effect similar to that of antibiotics. For growth performance, AMPs, bacteriophages, zymin, and oligosaccharides could replace antibiotics in weaned piglets; probiotics, plants, and plant extracts could replace antibiotics in growing pigs; and plants could replace antibiotics in finishing pigs. With regard to intestinal morphology, AMPs, plant extracts, and microelements were superior to antibiotics. Zymin and plant extracts were better than antibiotics at improving immunity.

**Table 4:** Assessment score of the effects of the interventions on outcomes

Interventions^a^	Bacteriostasis	Growth performance (weaned piglets)	Growth performance (growing pigs)	Growth performance (finishing pigs)	Intestinal morphology	Immunity	Sum (overall)	Sum (weaned piglets)	Sum (growing pigs)	Sum (finishing pigs)
Antibiotics	76.00	53.33	17.66	18.85	18.48	46.89	68.43	50.18	32.34	32.94
AMPs	**76.00**	**81.54**	0.00	0.00	**68.90**	0.00	55.26	**55.26**	14.49	14.49
Probiotics	0.00	46.85	**25.30**	15.92	4.18	0.00	44.45	23.84	13.07	8.38
Bacteriophages	0.00	**88.74**	0.00	0.00	0.00	0.00	44.37	44.37	0.00	0.00
Zymin	0.00	**55.33**	7.99	0.00	0.00	**64.74**	51.08	47.09	23.42	19.42
Plants	0.00	24.18	**19.42**	**27.42**	0.00	3.85	36.67	13.25	10.86	14.87
Plant extracts	32.00	15.44	**19.37**	7.55	**23.96**	**57.59**	44.05	30.59	**32.56**	26.65
Microelements	0.00	34.75	0.00	0.00	**69.23**	23.30	31.29	31.29	13.91	13.91
Oligosaccharides	19.00	**58.04**	0.00	0.00	0.00	19.39	36.73	36.73	7.72	7.72
Lysozyme	0.00	**55.07**	0.00	0.00	14.02	0.00	28.94	28.94	1.40	1.40
Organic acids	56.00	25.44	0.00	0.00	15.56	25.96	27.66	27.66	14.94	14.94
Basal diet	0.00	0.00	0.00	0.00	0.00	0.00	0.00	0.00	0.00	0.00

Bold type values for interventions indicates that their effect was superior or equal to that of antibiotics

^a^*AMPs* Antimicrobial peptides

## Discussion

**Fig. 4:**
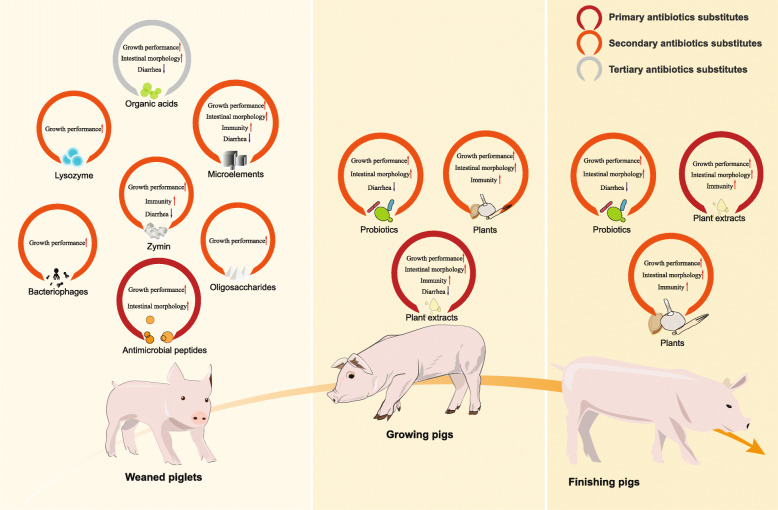
Summary of findings of meta-analyses

We used meta-analysis and NMA to define ASs. We detected the associations of 11 feed additives and 11 outcomes. The findings suggest that AMPs and plant extracts can be used as ASs for weaned piglets and growing pigs, respectively and that bacteriophages, zymin, plants, probiotics, oligosaccharides, lysozyme, and microelements can be regarded as secondary ASs (Fig. 4). Based on current data, the optimal AMPs, bacteriophage, lysozyme, microelements, oligosaccharides, organic acids, plants, plant extracts, probiotics, and zymin doses were 0.100%, 0.150%, 0.012%, 0.010%, 0.050%, 0.750%, 0.20%, 0.040%, 0.180%, and 0.100%, respectively.

To determine whether a feed additive is an eligible AS, it is necessary to measure its alternative effects on growth promotion, intestinal morphology improvement, bacteriostasis and immunity. Chief among these effects is growth promotion dependent on G/F. Feed additives enhance growth performance through improving intestinal morphology, reducing pernicious bacteria, reducing anti-nutritional factors, or improving nutrient digestibility. We did not investigate the effects of feed additives on the latter two mechanisms because antibiotics have not been reported to have these effects in the primary studies included in this meta-analysis. We measured intestinal morphology through V/C, as V/C is positively correlated with nutrient absorption capacity, such as that of carbohydrates and fatty acids [140]. We measured immunity through Ig and lymphocyte levels and the diarrhoea index/rate, which are the secondary phenotype outcomes. Consequently, antimicrobial and anti-inflammatory properties also contribute to promotion of growth performance. Bacteriostasis is measured by *in vitro* MIC experiments that are considered to provide reliable and stable results. Meanwhile, bacteriostasis effects of interventions are linked to antidiarrhoeal properties to some extent.

Our findings, together with mechanisms and possible speculations reported in articles, provide rational interpretations for growth promotion, immunity enhancement, and antidiarrhoeal properties for ASs. Interpretations of primary ASs are as follows. 1) AMPs can promote growth performance (ADG, ADFI, G/F) by improving intestinal morphology (V/C of the duodenum and ileum), nutrient digestibility, and antimicrobial activity [141]. AMPs can improve the duodenum and ileum by stimulating intestinal epithelial cell proliferation because AMP receptors may be rich in the duodenum and ileum [142]. AMPs are more likely to guarantee intestinal integrity and barrier function to protect from bacterial and toxin infections, which may be due to upregulation of the expression of tight junction proteins [102, 142, 143]. AMPs are critical components of the innate immune system, but evaluations of immune outcome associations were not conducted due to limitation by the lack of data. 2) Plant extracts can improve immunity (IgA, IgM, IgG, and lymphocyte levels) through their antimicrobial and anti-inflammatory properties [65]. Changing the microbiota and regulating intestinal permeability contribute to their antidiarrhoeal properties. The effects of plant extracts on growth performance (ADG and G/F) exhibit substantial heterogeneity because numerous plant extracts were included, and there is no feasible subgroup. Growth promotion associated with improving nutrient digestibility and amino acid metabolism also occurred [14]. Our pre-analyses identified a subgroup based on whether it is a plant essential oil, which cannot influence the substantial heterogeneity. A plant essential oil inhibits the opening of calcium channels and stimulates that of potassium channels in smooth muscles, which increases motility of the small intestine and produces a significant shortening of the food transit time [64, 144, 145]. However, several studies have suggested that a positive effect of plant essential oils seems to occur in challenged piglets rather than healthy piglets [64, 98]. Future meta-analyses should study the effects of plant extracts in pigs with different health statuses and the main sources of heterogeneity based on a feasible subgroup.

Interpretations of secondary ASs are as follows. 1) Zymin can improve growth performance (ADG and G/F), enhance immunity (IgA, IgM, IgG, and lymphocyte levels), and reduce and relieve diarrhoea. The reason for the above phenotype is that zymin can increase digestive enzyme activities and nutrient digestibility and decrease *Escherichia coli* and *Salmonella* populations [100]. 2) Lysozyme can increase growth performance (ADG, ADFI, and G/F), improve intestinal morphology (V/C of the duodenum and jejunum), and increase lymphocyte levels. Lysozyme increases protein deposition and decreases the turnover rate of intestinal epithelial cells [25, 85]. 3) Bacteriophages promote growth performance (ADG, ADFI, and G/F) through a reduction in coliforms and *Clostridium* [32]. 4) Plants can improve growth performance (ADG and G/F) and enhance immunity (IgG and lymphocyte levels). 4) Plants, specifically herbs, have antioxidant activity and pharmaceutical effects, providing additional benefits. Our previous meta-analysis indicated that fermented plants promoted growth performance and digestibility at all stages [8]. Additionally, fermented plants significantly improved marbling and decreased redness of the meat in finishing pigs but had no effect on lightness, yellowness, drip loss, and flavour [7]. 5) Probiotics can increase growth performance (ADG, ADFI, G/F) by improving nutrient digestibility and the microbiota structure, enhancing osmotic balance and reducing pernicious bacteria to contribute to the remission of diarrhoea [31]. Immunity promotion of probiotics was not observed; hence, the effect of probiotics on immunity is unclear. 6) Oligosaccharides can increase growth performance (ADG, ADFI, and G/F) and have no association with intestinal morphology, lymphocyte levels, or the diarrhoea rate. We speculate that oligosaccharides may increase nutrient digestibility, and the categories of oligosaccharides are related to the diarrhoea rate. 7) Microelements can increase growth performance (ADG, ADFI, and G/F), improve immunity (IgG levels), and reduce the risk of diarrhoea, which are linked to their bacteriostatic properties and improvement of the microbiota structure [74].

According to the results of the NMA, the effects of all feed additives investigated showed no significant difference from those of antibiotics on ADG, ADFI, IgG, and diarrhoea rate or index. The effects of bacteriophages are superior to those of antibiotics on weaned piglets’ G/F; the effects of microelements, plant extracts, and AMPs are superior to those of antibiotics on improvement of intestinal morphology; and the effects of plant extracts and zymin are superior to those of antibiotics on lymphocyte level enhancement. All of the ASs have potential uses in animal health. However, the high cost for many ASs, such as AMPs and bacteriophages, may be prohibitive for animal use. Future studies should further investigate high-efficiency bacterial engineering, purification technology, and the design of novel AMPs to expedite progress in reducing and alternating antibiotics. Commitment to substantial subsidies might be needed to incentivize development of ASs for animal health, in which their use could contribute to a reduction in antibiotic use [146].

A major strength of the present study is that we investigated all feed additives mentioned as ASs, which thus comprehensively demonstrated the effects of each feed additive on outcomes for which producers and animal nutritionists are interested. A major innovation is that we used a rational approach, the linear weighting sum model, to estimate the overall impact of each feed additive and antibiotics on pig health and growth. The limitation of the present study is that we failed to further investigate the main sources of heterogeneity of every AS and effects of combinational feed additives, such as combinations of plant essential oils and organic acids and those of prebiotics and probiotics. Some ASs were downgraded due to lack of some outcomes data. Future studies should investigate effects of feed additives on various aspects beyond growth performance.

## Conclusions

Here, we recommend supplementing 0.1% AMPs in the weaned stage, adding 0.04% plant extract in the growing stage and feeding 0.2% plants, especially fermented plants, in the finishing stage, which may have an approximate effect compared with antibiotics on all stages. Our research is the first to define and overall assess ASs through meta-analysis and NMA. Although further research should supplement unobserved data for a more comprehensive assessment, our research clearly and systematically investigates AS candidates. However, it is important to note that there is no single alternative to completely substitute antibiotics in feed, and a combination of different alternatives to antibiotics may be the most promising method to reduce or replace antibiotics in animal feeds. Future meta-analyses should further study the alternative effects of combinational feed additives.

## Supplementary Information

**Additional file 1: Table S1.** Search strategy. **Table S2.** Characteristics of studies. **Table S3.** Study quality assessment. **Table S4.** Minimal inhibitory concentration table (μg/mL). **Table S5.** Meta-analyses and subgroup analyses.

## Data Availability

All data generated or analysed during this study are included in this published article.

## Abbreviations

ADFI: Average daily feed intake
ADG: Average daily gain
AMPs: Antimicrobial peptides
ASs: Antibiotic substitutes
CI: Confidence interval
G/F: Gain: Feed ratio
SD: Standard deviation
SE: Standard error
SMD: Standard mean difference
SQANR: Study quality assessment on non-ruminants
V/C: Villus height:crypt depth ratio
