# Supplementary material for: Overall assessment of antibiotic substitutes for pigs: a set of meta-analyses
Source: J Anim Sci Biotechnol. 2021 Jan 7;12:3. doi: 10.1186/s40104-020-00534-2 (PMC7792336; doi:10.1186/s40104-020-00534-2)
Supplement: Supplementary file 1 — Additional file 1: Table S1. Search strategy. Table S2. Characteristics of studies. Table S3. Study quality assessment. Table S4. Minimal inhibitory concentration table (μg/mL). Table S5. Meta-analyses and subgroup analyses. [file 40104_2020_534_MOESM1_ESM.docx]

**Table S1.** Search strategy

| **Search** | **Query** | **Items found** |
| --- | --- | --- |
| #1 | Search ((((((((((((((((((((((((((((((((((((((((((((((probiotics[Title/Abstract]) OR Bacillus Coagulans[Title/Abstract]) OR yeast[Title/Abstract]) OR Lactobacillus plantarum[Title/Abstract]) OR Clostridium butyricum[Title/Abstract]) OR Lactobacillus rhamnosus GG[Title/Abstract]) OR anti-ETEC probiotic[Title/Abstract]) OR Lactobacillus acidophilus[Title/Abstract]) OR Clostridium butyricum[Title/Abstract]) OR Enterococcus faecium HDRsEf1[Title/Abstract]) OR Lactobacillus reuteri[Title/Abstract]) OR probiotic LSP 122[Title/Abstract]) OR Lactococcus lactis MG1363[Title/Abstract]) OR Brevibacillus laterosporus[Title/Abstract]) OR Bacillus laterosporus[Title/Abstract]) OR Bacillus licheniformis[Title/Abstract]) OR Bacillus subtilis[Title/Abstract]) OR Bifidobacterium bifidum[Title/Abstract]) OR Enterococcus faecalis[Title/Abstract]) OR Enterococcus faecium[Title/Abstract]) OR Enterococcus lactis[Title/Abstract]) OR Lactobacillus acidophilus[Title/Abstract]) OR Lactobacillus casei[Title/Abstract]) OR Lactobacillus delbrueckii subsp. Lactis[Title/Abstract]) OR Lactobacillus lactis[Title/Abstract]) OR Lactobacillus plantarum[Title/Abstract]) OR Pediococcus acidilactici[Title/Abstract]) OR Pediococcus pentosaceus[Title/Abstract]) OR Candida utilis[Title/Abstract]) OR Saccharomyces cerevisiae[Title/Abstract]) OR Rhodopseudomonas palustris[Title/Abstract]) OR Bifidobacterium infantis[Title/Abstract]) OR Bifidobacterium longum[Title/Abstract]) OR Bifidobacterium breve[Title/Abstract]) OR Bifidobacterium adolescentis[Title/Abstract]) OR Streptococcus thermophilus[Title/Abstract]) OR Lactobacillus reuteri[Title/Abstract]) OR Bifidobacterium animalis[Title/Abstract]) OR Aspergillus niger[Title/Abstract]) OR Aspergillus Oryzae[Title/Abstract]) OR Bacillus lentus[Title/Abstract]) OR Bacillus pumilus[Title/Abstract]) OR Lactobacillus cellobiosus[Title/Abstract]) OR Lactobacillus fermentum[Title/Abstract]) OR Lactobacillus delbrueckii subsp[Title/Abstract]) OR Bulgaricus[Title/Abstract]) OR Lactobacillus bulgaricus[Title/Abstract]) | 276534 |
| #2 | Search (((((((((((((((((((((((((((Oregano oil[Title/Abstract]) OR Thymol[Title/Abstract]) OR Eugenol[Title/Abstract]) OR Carvacrol[Title/Abstract]) OR Cinnanmaldehyde[Title/Abstract]) OR Olive oil[Title/Abstract]) OR Macleaya cordata Extract[Title/Abstract]) OR scutellaria baicalensis extract[Title/Abstract]) OR Psyllium, Astragalus[Title/Abstract]) OR Echinacea[Title/Abstract]) OR herbal medicines[Title/Abstract]) OR herb[Title/Abstract]) OR curcumin[Title/Abstract]) OR inulin[Title/Abstract]) OR seaweed extracts[Title/Abstract]) OR Lentinan[Title/Abstract]) OR Saccharicterpenin[Title/Abstract]) OR Medicago sativa Extract[Title/Abstract]) OR Eucommia Ulmoides Extract[Title/Abstract]) OR Epimedium Extract[Title/Abstract]) OR Extrat of Perilla frutescens seed[Title/Abstract]) OR Astragalus polysaccharide[Title/Abstract]) OR algal polysaccharides[Title/Abstract]) OR ganoderan[Title/Abstract]) OR Polyporus polysaccharide[Title/Abstract]) OR cinnamon[Title/Abstract]) OR garlic[Title/Abstract]) OR extract[Title/Abstract]) | 252877 |
| #3 | Search lysozyme | 31975 |
| #4 | Search (((((((((((((((organic acid[Title/Abstract]) OR Capric Acid[Title/Abstract]) OR Calcium Acetate[Title/Abstract]) OR Potassium Diformate[Title/Abstract]) OR Formic Acid[Title/Abstract]) OR Acetic Acid[Title/Abstract]) OR Propionic Acid[Title/Abstract]) OR Butyric Acid[Title/Abstract]) OR Lactic Acid[Title/Abstract]) OR Benzoic Acid[Title/Abstract]) OR Sorbic Acid[Title/Abstract]) OR Fumaric Acid[Title/Abstract]) OR Citric Acid[Title/Abstract]) OR Tartaric Acid[Title/Abstract]) OR Malic Acid[Title/Abstract]) OR Phosphoric Acid[Title/Abstract]) | 122849 |
| #5 | Search ((((((antimicrobial peptides[Title/Abstract]) OR antibacterial peptides[Title/Abstract]) OR cathelicidin[Title/Abstract]) OR Antibacterial peptide[Title/Abstract]) OR Prophenin, Protegrin[Title/Abstract]) OR pBD-1[Title/Abstract]) OR Cecropin[Title/Abstract]) | 13278 |
| #6 | Search ((((((((((((((chitosan[Title/Abstract]) OR Alginate oligosaccharide[Title/Abstract]) OR Pectic Oligosaccharide[Title/Abstract]) OR xylo-oligosaccharide[Title/Abstract]) OR Low-molecular-weight Chitosan[Title/Abstract]) OR Galactomanno-oligosaccharides[Title/Abstract]) OR 尾-Glucan[Title/Abstract]) OR Fructo-oligosaccharides[Title/Abstract]) OR Manno-oligosaccharides[Title/Abstract]) OR Galacto-oligosaccharides[Title/Abstract]) OR Chitosan-oligosaccharide[Title/Abstract]) OR 1,3-D-glucan[Title/Abstract]) OR N,O-carboxymethyl chitosan[Title/Abstract]) OR peptidoglycan[Title/Abstract]) OR lipopolysaccharide[Title/Abstract]) | 119980 |
| #7 | Search (((((((((((((Bromelain[Title/Abstract]) OR Amylase[Title/Abstract]) OR Galactosidase[Title/Abstract]) OR Cellulase[Title/Abstract]) OR 尾-Glucanase[Title/Abstract]) OR Glucose Oxidase[Title/Abstract]) OR Lipase[Title/Abstract]) OR Maltase[Title/Abstract]) OR Mannanase[Title/Abstract]) OR Pectinase) OR Phytase) OR Protease) OR Keratinase) OR Xylanase) | 600221 |
| #8 | Search (((((bacteriophages[Title/Abstract]) OR wild-type bacteriophages[Title/Abstract]) OR AmpliPhi[Title/Abstract]) OR Engineered bacteriophages[Title/Abstract]) OR Phico Therapeutics[Title/Abstract]) OR Phage lysins[Title/Abstract]) | 8365 |
| #9 | Search ((Zinc oxide nanoparticles[Title/Abstract]) OR aluminosilicate[Title/Abstract]) OR Selenium[Title/Abstract]) | 29449 |
| #10 | Search (((((((((Casein glycomacropeptide[Title/Abstract]) OR fusion peptide bovine lactoferricin-lactoferrampin[Title/Abstract]) OR CpG oligodeoxynucleotide[Title/Abstract]) OR beta-defensin-2[Title/Abstract]) OR Bee pollen[Title/Abstract]) OR bursa extracts[Title/Abstract]) OR gamma globulin[Title/Abstract]) OR heat shock protein[Title/Abstract]) OR poly IC[Title/Abstract]) OR glycyrrhizin[Title/Abstract]) | 40381 |
| #11 | Search ((#1 OR #2 OR #3 OR #4 OR #5 OR #6 OR #7 OR #8 OR #9 OR #10)) | 1402707 |
| #12 | Search (((((((pig) OR weaned piglet) OR weaned pig) OR nursery) OR growing pig) OR finishing pig)))) AND (((#1 OR #2 OR #3 OR #4 OR #5 OR #6 OR #7 OR #8 OR #9 OR #10))))) NOT review[Publication Type] Filters: Journal Article; Abstract; Publication date from 2000/01/01 to 2019/04/31; Other Animals Sort by: [pubsolr12] | 12053 |
| #13 | Search ((((#1 OR #2 OR #3 OR #4 OR #5 OR #6 OR #7 OR #8 OR #9 OR #10)))) AND Minimum inhibitory concentration Filters: Journal Article; Abstract; Publication date from 2000/01/01 to 2019/04/31; Other Animals | 4256 |

**Table S2.** Characteristics of studies

| Study | Country | Growth stage | Add amount | Treatment | Control group | Sample size | Initial BW, kg | Duration or final BW | Outcomes |
| --- | --- | --- | --- | --- | --- | --- | --- | --- | --- |
| Biagi et al. [1] | Italy | Weaned piglets | 0.3%, 0.6% | Gluconic acid | Basal diet | 48 | 7.44 | 6 weeks | ADG, ADFI, GF |
| Biagi et al. [2] | Italy | Weaned piglets | 0.1%, 0.2%, 0.4% | Sodium butyrate | Basal diet | 48 | 6.68 | 6 weeks | ADG, ADFI, GF |
| Bosi et al. [3] | Italy | Weaned piglets | NA | NA | Basal diet & antibiotics | 36 | 6.66 | 4 weeks | ADG, ADFI, GF |
| Chen et al. [4] | China | Weaned piglets | 0.2% , 0.5% | Benzoic acid | Basal diet | 30 | 6.73 | 6 weeks | ADG, ADFI, GF, JV/C |
| Chu et al. [5] | *Korea* | Finishing pigs | 0.30% | Bamboo charcoal | Basal diet & antibiotics | 108 | 79 | 6 weeks | ADG, ADFI, IgA, IgM, IgG |
| Chu et al. [5] | *Korea* | Finishing pigs | 0.30% | Bamboo vinegar | Basal diet & antibiotics | 108 | 78.61 | 6 weeks | ADG, ADFI, IgA, IgM, IgG |
| Fang et al. [6] | China | Weaned piglets | 0.10% | Sodium butyrate | Basal diet | 100 | 8 | 21 days | ADG, ADFI, GF |
| Kaewtapee et al. [7] | Thailand | Growing pigs | 0.05%, 0.1% | LMA | Basal diet | 24 | 18.9 | 6 weeks | ADG, ADFI, V/C |
| Kluge et al. [8] | Germany | Weaned piglets | 0.5%, 1% | Benzoic acid | Basal diet | 54 | 7.5 | 35 days | ADG, ADFI, GF |
| Li et al. [9] | China | Weaned piglets | 0.2%, 0.3%, 0.5% | Organic acids | Basal diet & antibiotics | 240 | 7.2 | 28 days | ADG, ADFI, GF, V/C, diarrhoea index |
| Luo et al. [10] | China | Weaned piglets | 0.05%, 0.2%, 0.4% | Sorbic acid | Basal diet | 240 | 6.86 | 35 days | ADG, ADFI, GF |
| Mair et al. [11] | Austria | Weaned piglets | 0.40% | Inulin | Basal diet | 23 | 9.18 | 28 days | ADG, ADFI |
| Mair et al. [11] | Austria | Weaned piglets | 1×10^9^ CFU/kg | Probiotics | Basal diet | 23 | 9.18 | 28 days | ADG, ADFI |
| Omogbenigun et al. [12] | Canada | Weaned piglets | 500 U/kg | Microbial phytase | Basal diet | 48 | 6.41 | 4 weeks | ADG, ADFI, GF |
| Upadhaya et al. [13] | South Korea | Growing pigs | 0.05% | Β-mannanase | Basal diet | 70 | 25.1 | 6 weeks | ADG, ADFI, GF |
| Walsh et al. [14] | America | Weaned piglets | 0.2%-0.4% | 0.4% organic acid d 0 to 7 & 0.2% inorganic acid d 7 to 34 | Basal diet & antibiotics | 204 | 5.5 | 34 days | ADG, ADFI, GF |
| Yang et al. [15] | China | Weaned piglets | 0.10% | Plant essential oils and organic acids | Basal diet & antibiotics | 300 | 6.11 | 28 days | ADG, ADFI, GF,  diarrhoea rate, IgA, IgM, IgG |
| Yen et al. [16] | China | Weaned piglets | NA | NA | Basal diet & antibiotics | 264 | 6.1 | 2 weeks | ADG, ADFI, GF |
| Espinosa et al. [17] | Urbana | Weaned piglets | 0.015% | Cu | Basal diet | 40 | 6.8 | 4 weeks | ADG, ADFI, GF |
| Espinosa et al. [17] | Urbana | Weaned piglets | 0.01%, 0.02% | Cu | Basal diet | 66 | 9.89 | 4 weeks | ADG, ADFI, GF |
| Han et al. [18] | China | Weaned piglets | 0.005%, 0.01% | Chitosan (CS)-Zn | Basal diet | 150 | 7.2 | 28 days | V/C |
| Han et al. [18] | China | Weaned piglets | 0.01% | ZnSO4 |  |  |  |  | V/C |
| Han et al. [18] | China | Weaned piglets | 0.30% | ZnO |  |  |  |  | V/C |
| Jlali et al. [19] | France | Growing pigs | 0.00001%, 0.00003% | Sodium selenite | Basal diet | 112 | 26.73 | 32 days | ADG, ADFI, GF |
| Jlali et al. [19] | France | Growing pigs | 0.00001%, 0.00003% | Se-enriched yeast | Basal diet |  |  |  | ADG, ADFI, GF |
| Jlali et al. [19] | France | Growing pigs | 0.00001%, 0.00003% | 2-Hydroxy-4-methylselenobutanoic acid | Basal diet |  |  |  | ADG, ADFI, GF |
| Li et al. [20] | China | Weaned piglets | 0.012% | Zn（ZnO） | Basal diet | 96 | 8.04 | 45 days | ADG, GF, IgG |
| Li et al. [20] | China | Weaned piglets | 0.012% | Zn（Zn-methionine） | Basal diet |  |  |  |  |
| Li et al. [20] | China | Weaned piglets | 0.012% | Zn（nano-Zn） | Basal diet |  |  |  |  |
| Marcolla et al. [21] | Brazil | Finishing pigs | 0.00004% | Chromium | Basal diet | 32 | 70.21 | 115 kg | ADG, ADFI, GF |
| Mateo et al. [22] | America | Growing pigs  Finishing pigs | 0.00001%, 0.00002%, 0.00003% | Organic source (Se-yeast; selenosource-AF, Diamond V Mills Inc., Cedar Rapids, IA) | Basal diet | 180 | 34.4 | 129.9kg | ADG, ADFI, GF |
| Mateo et al. [22] | America | Finishing pigs | 0.00003% | Sodium selenite | Basal diet |  |  |  | ADG, ADFI, GF |
| Choi et al. [23] | Korea | Weaned piglets | 0.30% | Probiotic LT | Basal diet & antibiotics | 288 | 6.43 | 28 days | ADG, ADFI, GF |
| Choi et al. [23] | Korea | Weaned piglets | 0.30% | Probiotic HT | Basal diet & antibiotics |  |  |  | ADG, ADFI, GF |
| Chu et al. [24] | Korea | Weaned piglets | 0.20% | Probiotics from anaerobic bacteria with prebiotics | Antibiotics | 150 | 11.09 | 15 days | ADG, ADFI, GF |
| Chu et al. [24] | Korea | Weaned piglets | 0.20% | Probiotics from anaerobic yeast with prebiotics | Antibiotics |  |  |  | ADG, ADFI, GF |
| Chu et al. [24] | Korea | Weaned piglets | 0.20% | Probiotics from anaerobic mould with prebiotics | Antibiotics |  |  |  | ADG, ADFI, GF |
| Chu et al. [24] | Korea | Weaned piglets | 0.20% | Probiotics compounded anaerobic bacteria, yeast and mould with prebiotics | Antibiotics |  |  |  | ADG, ADFI, GF |
| Hancox et al. [25] | France | Weaned piglets | 3.3 × 10^9^ CFU | *Saccharomyces cerevisiae* var. boulardii | Basal diet | 46 | 1.4 | 7 days | ADG, diarrhoea index |
| Kim et al. [26]] | Korea | Weaned piglets | 0.2%,0.4% | *L. acidophilus* strain 30SC | Basal diet & antibiotics | 48 | 10.75 | 28 days | ADG, ADFI, GF |
| Lan et al. [27] | Korea | Weaned piglets | 0.01%, 0.03%, 0.06%, 0.1% | Multistrain probiotics | Basal diet | 125 | 7.26 | 42 days | ADG, ADFI, GF |
| Lei and Kim [28] | Korea | Finishing pigs | 0.1%, 0.2% | *Phaffia rhodozyma* | Basal diet | 96 | 58.61 | 10 weeks | ADG, ADFI, GF |
| Li et al. [29] | China | Weaned piglets | 4×10^6^ , 9×10^6^ , 2.6×10^7^, 5.1×10^7^  CFU/g | *Saccharomyces cerevisiae* | Basal diet | 90 | 7.2 | 28 days | ADG, ADFI, GF |
| Li et al. [29] | China | Weaned piglets | 3.0 × 10^7^ cfu/g | *Saccharomyces cerevisiae* | Basal diet | 48 | 7.64 | 28 days | ADG, ADFI, GF |
| Liu et al. [30] | China | Growing pigs | 6.0× 10^7^ CFU/kg | *B. subtilis* endospores and *S. cerevisiae* endospores | Basal diet | 50 | 24.88 | 6 weeks | ADG, ADFI, GF |
| Shen et al. [31] | China | Growing pigs | 0.25%, 0.5% | Yeast culture | Basal diet & antibiotics | 128 | 7.5 | 3 weeks | ADG, ADFI, GF |
| Shen et al. [31] | China | Growing pigs | 0.50% | Yeast culture | Basal diet & antibiotics | 24 | 5.8 | 3 weeks | ADG, ADFI, GF |
| van Heugten et al. [32] | America | Growing pigs | 1.6 × 10^7^ CFU/g | Yeast | Basal diet | 78 | 5.9 | 28 days | ADG, ADFI, GF |
| Hu et al. [33] | China | Weaned piglets | 0.0050% | Low-molecular-weight chitosan | Basal diet | 40 | 6.37 | 4 weeks | ADG,ADFI,G/F, diarrhoea rate, V/C |
| Manzanilla et al. [34] | Spain | Weaned piglets | 0.30% | Sodium butyrate | Basal diet & antibiotics | 24 | 6 | 2 weeks | ADG, ADFI, G/F, V/C |
| Manzanilla et al. [34] | Spain | Weaned piglets | 0.03% | Plant extract combination | Basal diet & antibiotics | 24 | 6 | 2 weeks | ADG, ADFI, G/F, V/C |
| Pan et al. [35] | China | Growing pigs | 0.01%, 0.05% | α-Galactosidase Preparation | Basal diet | 72 | 15.77 | 3 weeks | ADG, G/F,  diarrhoea index |
| Partanen et al. [36] | Finland | Weaned piglets | 0.80% | Formic acid | Basal diet & antibiotics | 60 | 9.17 | 18.22 kg | ADG, G/F,  diarrhoea index |
| Partanen et al. [36] | Finland | Weaned piglets | 0.80% | Mixture of formic acid, propionic acid, and potassium sorbate | Basal diet & antibiotics | 60 | 9.17 | 18.22 kg | ADG, G/F,  diarrhoea index |
| Partanen et al. [36] | Finland | Weaned piglets | 0.80% | Mixture of formic acid, propionic acid, and sodium benzoate | Basal diet & antibiotics | 60 | 9.17 | 18.22 kg | ADG, G/F,  diarrhoea index |
| Partanen et al. [36] | Finland | Weaned piglets | 0.80% | Formic acid in a diatomaceous earth carrier | Basal diet & antibiotics | 60 | 9.17 | 18.22 kg | ADG, G/F |
| Partanen et al. [36] | Finland | Growing pigs | 0.60% | Formic acid | Basal diet & antibiotics | 91 | 18.25 | 46.78 kg | ADG, G/F |
| Partanen et al. [36] | Finland | Growing pigs | 0.60% | Mixture of formic acid, propionic acid, and potassium sorbate | Basal diet & antibiotics | 91 | 18.25 | 46.78 kg | ADG, G/F |
| Partanen et al. [36] | Finland | Growing pigs | 0.60% | Mixture of formic acid, propionic acid, and sodium benzoate | Basal diet & antibiotics | 84 | 18.25 | 46.78 kg | ADG, G/F |
| Partanen et al. [36] | Finland | Growing pigs | 0.60% | Formic acid in a diatomaceous earth carrier | Basal diet & antibiotics | 81 | 18.25 | 46.78 kg | ADG, G/F |
| Partanen et al. [36] | Finland | Finishing pigs | 0.60% | Formic acid | Basal diet & antibiotics | 91 | 46.78 | 103.03 kg | ADG, G/F |
| Partanen et al. [36] | Finland | Finishing pigs | 0.60% | Mixture of formic acid, propionic acid, and potassium sorbate | Basal diet & antibiotics | 91 | 46.78 | 103.03 kg | ADG, G/F |
| Partanen et al. [36] | Finland | Finishing pigs | 0.60% | Mixture of formic acid, propionic acid, and sodium benzoate | Basal diet & antibiotics | 84 | 46.78 | 103.03kg | ADG, G/F |
| Partanen et al. [36] | Finland | Finishing pigs | 0.60% | Formic acid in a diatomaceous earth carrier | Basal diet & antibiotics | 81 | 46.78 | 103.03 kg | ADG, G/F |
| Qian et al. [37] | China | Weaned piglets | 0.01% | ZnSO_4_ | Basal diet | 60 | 6.11 | 30 days | ADG, ADFI, G/F, Diarrhoea rate |
| Qian et al. [37] | China | Weaned piglets | 0.01% | Chitosan-Zn chelate | Basal diet | 60 | 6.11 | 31 days | ADG, ADFI, G/F, Diarrhoea rate |
| Qian et al. [37] | China | Weaned piglets | 0.01% | ZnSO_4_ mixed with chitosan | Basal diet | 60 | 6.11 | 32 days | ADG, ADFI, G/F, Diarrhoea rate |
| van der Peet-Schwering et al. [38] | Netherlands | Weaned piglets | 0.13% | Yeast culture | Basal diet & antibiotics | 360 | 7.8 | 5 weeks | ADG, ADFI, G/F, Lymphocyte, Jejunum V/C |
| van der Peet-Schwering et al. [38] | Netherlands | Weaned piglets | 0.33% | Yeast Culture and cell wall product | Basal diet & antibiotics | 360 | 7.8 | 5 weeks | ADG, ADFI, G/F, Lymphocyte, Jejunum V/C |
| Van Nevel et al. [39] | Belgium | Weaned piglets | 1% | Guar gum | Basal diet | 12 | 9.45 | 12 days | Jejunum V/C |
| Van Nevel et al. [39] | Belgium | Weaned piglets | 1% | Locust bean gum | Basal diet | 12 | 9.45 | 12 days | Jejunum V/C |
| Walsh et al. [40] | Ireland | Weaned piglets | 0.03% | 5 to 10 kDa chitooligosaccharide | Basal diet | 14 | 9.1 | 8 days | V/C |
| Walsh et al. [40] | Ireland | Weaned piglets | 0.03% | 10 to 50 kDa chitooligosaccharide | Basal diet | 14 | 9.1 | 8 days | V/C |
| Walsh et al. [40] | Ireland | Weaned piglets | 0.03% | 50 to 100 kDa chitooligosaccharide | Basal diet | 14 | 9.1 | 8 days | V/C |
| Yeh et al. [41] | China | Weaned piglets | 0.30% | Chinese herbal medicine complex | Basal diet & antibiotics | 36 | 10 | 39.63 kg | ADG, ADFI, G/F,IgG |
| Zhao et al. [42] | China | Weaned piglets | 0.01% | *Forsythia suspensa* extract | Basal diet | 72 | 8.24 | 4 weeks | ADG, ADFI, G/F, Diarrhoea rate |
| Zhao et al. [42] | China | Weaned piglets | 0.02% | Chitooligosaccharide | Basal diet | 72 | 8.24 | 4 weeks | ADG, ADFI, G/F, Diarrhoea rate |
| Lee et al. [43] | China | Weaned piglets | 0.60% | Organic acids | Basal diet | 34 | 7.23 | 46 days | ADG, ADFI, G/F, IgA, IgM, IgG |
| Lee et al. [43] | China | Weaned piglets | 0.10% | Nucleotides | Basal diet | 34 | 7.23 | 46 days | ADG, ADFI, G/F, IgA, IgM, IgG |
| Lee et al. [43] | China | Weaned piglets | 0.60% | Organic acids | Basal diet | 8 | 7.23 | 21 days | Jejunum V/C, Ileum V/C, IgA, IgM, IgG |
| Lee et al. [43] | China | Weaned piglets | 0.10% | Nucleotides | Basal diet | 8 | 7.23 | 21 days | Jejunum V/C, Ileum V/C, IgA, IgM, IgG |
| Superchi et al. [44] | Italy | Weaned piglets | 0.10% | Yeast extract nucleotides | Basal diet | 108 | 3.18 | 47 days | ADG, ADFI, G/F |
| Waititu et al. [45] | Canada | Weaned piglets | 0.10% | Nucleotide-rich yeast extract | Basal diet | 42 | 7.11 | 14 days | ADG, ADFI, G/F, Ileum V/C |
| Wang et al. [46] | China | Weaned piglets | 0.10% | Lactoferrin | Basal diet & antibiotics | 90 | 7.05 | 15 days | ADG, ADFI, G/F,Jejunum V/C |
| Yi et al. [47] | China | Weaned piglets | 5×10^10^ CFU/kg | *Lactobacillus reuteri* LR1 | Basal diet & antibiotics | 144 | 6.49 | 14 days | ADG, ADFI, G/F, V/C |
| Chen et al. [48] | China | Weaned piglets | 0.025%, 0.05%, 0.1% | Chlorogenic acid | Basal diet | 200 | 7.65 | 14 days | ADG, ADFI, G/F |
| Chen et al. [48] | China | Weaned piglets | 0.1% | Chlorogenic acid | Basal diet | 24 | 6.83 | 14 days | ADG, ADFI, G/F, diarrhoea index |
| Clarke et al. [49] | Ireland | Weaned piglets | 0.01% | β-Glucanase and β-xylanase enzyme | Basal diet | 64 | 11.7 | 35 days | ADG, ADFI, G/F |
| Davis et al. [50] | America | Weaned piglets | 0.30% | Phosphorylated mannans | Basal diet | 32 | 5.7 | 21 days | ADG, ADFI, G/F, Lymphocyte |
| Fang et al. [51] | China and Singapore | Growing pigs | 0.025%, 0.05% | Xylanase | Basal diet | 42 | 27.8 | 68 kg | ADG, ADFI, G/F |
| Jo et al. [52] | Korea | Finishing pigs | 0.05% | β-Mannanase | Basal diet | 96 | 55.6 | 28 days | ADG, ADFI, G/F |
| Jo et al. [52] | Korea | Finishing pigs | 0.05% | α-Amylase + β-mannanase | Basal diet | 96 | 55.6 | 28 days | ADG, ADFI, G/F |
| Jo et al. [52] | Korea | Finishing pigs | 0.05% | β-Mannanase + protease | Basal diet | 96 | 55.6 | 28 days | ADG, ADFI, G/F |
| Jo et al. [52] | Korea | Finishing pigs | 0.05% | α-Amylase + β-mannanase + protease | Basal diet | 96 | 55.6 | 28 days | ADG, ADFI, G/F |
| Lan et al. [53] | Korea | Weaned piglets | 0.005%, 0.01% | Xylanase | Basal diet | 150 | 7.85 | 42 days | ADG, ADFI, G/F |
| Molist et al. [54] | Spain | Weaned piglets | 0.30% | Zinc oxide | Basal diet | 32 | 6.7 | 12 days | ADG, ADFI, G/F, diarrhoea rate |
| Omogbenigun et al. [55] | Canada | Weaned piglets |  | Cellulase, galactanase, and mannanase | Basal diet | 12 | 7 | 4 weeks | ADG, ADFI, G/F |
| Omogbenigun et al. [55] | Canada | Weaned piglets |  | Cellulose and pectinase | Basal diet | 12 | 7 | 4 weeks | ADG, ADFI, G/F |
| Omogbenigun et al. [55] | Canada | Weaned piglets |  | Cellulase, galactanase, mannanase, and pectinase | Basal diet | 12 | 7 | 4 weeks | ADG, ADFI, G/F |
| Owusu-Asiedu et al. [56] | UK | Weaned piglets | 0.005%,0.01%,0.02% | Xylanase and β-glucanase blend | Basal diet | 192 | 6.5 | 42 days | ADG, ADFI, G/F |
| Shan et al. [57] | China | Weaned piglets | 0.10% | Lactoferrin | Basal diet & antibiotics | 90 | 7.05 | 30 days | ADG, ADFI, G/F, diarrhoea rate, IgA, IgM, IgG, Lymphocyte |
| Taylor et al. [58] | UK | Weaned piglets | 8000, 16 000 or 32 000 BXU/kg | Xylanase | Basal diet | 512 | 14.2 | 5 weeks | ADG, ADFI, G/F |
| Vahjen et al. [59] | Germany | Weaned piglets | 0.01% | Xylanase-, 1,3-1,4-ß-glucanase-, 1,4-ß-mannanase-, 1,4-ß-galactanase-, and cellulase | Basal diet | 40 | NA | 5 weeks | ADG, ADFI, G/F |
| Vahjen et al. [59] | Germany | Weaned piglets | 0.02% | Xylanase | Basal diet | 40 | NA | 5 weeks | ADG, ADFI, G/F |
| Walk et al. [60] | UK and Thailand | Weaned piglets | 2500 FTU | Microbial phytase | Basal diet | 96 | 7.1 | 42 days | ADG, ADFI, G/F |
| Walk et al. [60] | UK and Thailand | Weaned piglets | 0.175%, 0.35% | Zinc oxide | Basal diet | 144 | 7.1 | 42 days | ADG, ADFI, G/F |
| Zhang et al. [61] | China and Canada | Weaned piglets | 0.01%, 0.015%, 0.025%, 0.035% | Exogenous multi-enzyme | Basal diet | 200 | 9.59 | 30 days | ADG, ADFI, G/F, Diarrhoea index |
| Zhang et al. [62] | China | Finishing pigs | 0.03% | α-Galactosidase + xylanase | Basal diet | 216 | 44.7 | 43 days | ADG, ADFI, G/F |
| Long et al. [63] | China | Weaned piglets | 0.003%, 0.006%, 0.009%, 0.012%, | Lysozyme | Basal diet & antibiotics | 150 | 7.32 | 28 days | ADG, ADFI, G/F, Lymphocyte,V/C |
| Oliver and Wells [64] | America | Weaned piglets | 0.01% | Lysozyme | Basal diet & antibiotics | 192 | 7.8 | 29 days | ADG, ADFI, G/F |
| Oliver et al. [65] | America | Weaned piglets | 0.01% | Lysozyme | Basal diet & antibiotics | 300 | 8.6 | 4 weeks | ADG, ADFI, G/F |
| Hosseindoust et al. [66] | USA | Weaned piglets | 0.10% | Bacteriophages | Basal diet | 100 | 8.08 | 35 days | ADG, ADFI, G/F, V/C |
| Kim et al. [67] | Korea | Weaned piglets | 0.1%, 0.15% | Bacteriophages | Basal diet | 150 | 7.77 | 36 days | ADG, ADFI, G/F, V/C, IgA, IgM, IgG |
| Kim et al. [67] | Korea | Weaned piglets | 0.30% | Multi-strain probiotics | Basal diet | 100 | 7.76 | 37 days | ADG, ADFI, G/F, V/C, IgA, IgM, IgG |
| Begum et al. [68] | Korea | Weaned piglets | 0.1%, 0.2% | Fenugreek seed extract | Basal diet | 135 | 7.96 | 42 days | ADG, ADFI, G/F, Lymphocyte, IgG |
| Chen et al. [69] | China | Weaned piglets | 0.30% | *Macleaya cordata* extract | Basal diet | 20 | 6.52 | 21 days | ADG, ADFI, G/F, V/C |
| Cheng et al. [70] | China | Growing pigs | 0.025% | Oregano essential oil | Basal diet & antibiotics | 36 | 29.64 | 63.61 kg | ADG, ADFI, G/F, V/C |
| Cheng et al. [70] | China | Finishing pigs | 0.025% | Oregano essential oil | Basal diet & antibiotics | 36 | 63.61 | 108.70 kg | ADG, ADFI, G/F, V/C |
| Di Giancamillo et al. [71] | Italy | Weaned piglets | 0.0005% | Verbascoside | Basal diet | 16 | 10.9 | 30 days | ADG, G/F |
| Ilsley et al. [72] | UK | Weaned piglets | 0.100% | Quillaja | Basal diet | 96 | 7.7 | 21 days | ADG, ADFI, G/F, IgA, IgG |
| Ilsley et al. [72] | UK | Weaned piglets | 0.020% | Curcumin | Basal diet | 96 | 7.7 | 21 days | ADG, ADFI, G/F, IgA, IgG |
| Jeong and Kim [73] | Korea | Growing pigs | 0.05%, 0.1%, 0.2% | Fermented medicinal plants | Basal diet & antibiotics | 150 | 25.5 | 6 weeks | ADG, ADFI, G/F, Lymphocyte |
| Kang et al. [74] | Korea | Finishing pigs | 0.03% | Herb extracts | Basal diet | 20 | 49.46 | 69 days | ADG, ADFI, G/F |
| Kang et al. [74] | Korea | Finishing pigs | 0.10% | Aminolevulinic acid. | Basal diet | 20 | 49.46 | 69 days | ADG, ADFI, G/F |
| Lei et al. [75] | Korea | Growing pigs | 0.05% | Plant extract YGF251 | Basal diet | 48 | 24.72 | 6 weeks | ADG, ADFI, G/F |
| Li and Kim [28] | China | Growing pigs | 0.05%, 0.1% | *Saccharomyces cerevisiae* extract  Poplar propolis ethanol extract | Basal diet | 105 | 24.91 | 6 weeks | ADG, ADFI, G/F, Lymphocyte, IgG |
| Manzanilla et al. [76] | France | Weaned piglets | 0.015%, 0.03% | Plant extracts | Basal diet | 108 | 8.1 | 21 days | ADG, ADFI, G/F |
| Manzanilla et al. [76] | France | Weaned piglets | 0.50% | Formic acid | Basal diet | 108 | 8.1 | 21 days | ADG, ADFI, G/F |
| Nowak et al. [77] | Poland | Weaned piglets | 0.02% | Plant extracts | Basal diet | 24 | 12.9 | 36.8 kg | ADG, ADFI, G/F |
| Schone et al. [78] | Germany | Weaned piglets | 0.10% | Fennel essential oils | Basal diet | 30 | 7 | 4 weeks | ADG, ADFI, G/F |
| Schone et al. [78] | Germany | Weaned piglets | 0.10% | Caraway essential oils | Basal diet | 30 | 7 | 4 weeks | ADG, ADFI, G/F |
| Wang et al. [79] | China | Weaned piglets | 0.005%, 0.01%, 0.02% | *Piper sarmentosum* extract | Basal diet | 80 | 6.43 | 4 weeks | ADG, ADFI, G/F |
| Yan and Kim [80] | Korea | Weaned piglets | 0.05%, 0.1%, 0.2% | Fermented garlic powder | Basal diet | 144 | 5.5 | 5 weeks | ADG, ADFI, G/F, Lymphocyte |
| Yan et al. [81] | Korea | Finishing pigs | 0.1%, 0.2%, 0.4% | Fermented garlic powder | Basal diet & antibiotics | 100 | 50.7 | 6 weeks | ADG, ADFI, G/F, Lymphocyte, IgG |
| Yan et al. [81] | Korea | Finishing pigs | 0.1%, 0.2%, 0.4% | Fermented garlic powder | Basal diet & antibiotics | 100 | 78.2 | 6 weeks | ADG, ADFI, G/F, Lymphocyte, IgG |
| Yin et al. [82] | China | Weaned piglets | 0.10% | Astragalus polysaccharide | Basal diet & antibiotics | 60 | 7.35 | 4 weeks | ADG, ADFI, G/F |
| Cutler et al. [83] | America | Weaned piglets | 0.0011%, 0.00165% | Colicin E1 | Basal diet | 24 | 23 days | NA | ADG, ADFI, G/F |
| Tang et al. [84] | China | Weaned piglets | 0.01% | Lactoferrin | Basal diet | 30 | 5.42 | 3 weeks | ADG, ADFI, G/F |
| Wu et al. [85] | China | Weaned piglets | 0.04% | Cecropin AD | Basal diet & antibiotics | 24 | 6.76 | 19 days | ADG, ADFI ,G/F, V/C |
| Xiao et al. [86] | China | Weaned piglets | 0.40% | Composite antimicrobial peptides | Basal diet | 14 | NA | 30 days | Ileum VC, Jejunum VC |
| Xiao et al. [87] | China | Weaned piglets | 0.40% | Composite antimicrobial peptides | Basal diet | 14 | 11.9 | 30 days | ADG, ADFI, G/F |
| Yoon et al. [88] | Korea | Weaned piglets | 0.006%, 0.009% | Antimicrobial peptide-A3 | Basal diet & antibiotics | 60 | 5.74 | 28 days | ADG, ADFI,G/F, V/C |
| Yoon et al. [89] | Korea | Weaned piglets | 0.004%, 0.006% | Antimicrobial peptide-P5 | Basal diet & antibiotics | 240 | 6.22 | 28 days | ADG, ADFI, G/F |

BW, body weight; LMA, liquid DL-methionine hydroxy analogue free acid; VC, Villus height: Crypt depth;

**Table S3.** Study quality assessment

| Study | With-in group differences | Multiple reports | Sample size | Rationality of experimental design | Completeness of experimental information | Score | Quality |
| --- | --- | --- | --- | --- | --- | --- | --- |
| Biagi et al. [1] | 0 | 5 | 2 | 4 | 4 | 15 | Moderate |
| Biagi et al. [2] | 0 | 5 | 2 | 4 | 4 | 15 | Moderate |
| Bosi et al. [3] | 0 | 5 | 2 | 4 | 2 | 13 | Low |
| Chen et al. [4] | 0 | 5 | 2 | 4 | 5 | 16 | Moderate |
| Chu et al. [5] | 0 | 5 | 5 | 4 | 5 | 19 | Moderate |
| Fang et al. [6] | 5 | 5 | 4 | 4 | 5 | 23 | High |
| Kaewtapee et al. [7] | 0 | 5 | 1 | 4 | 4 | 14 | Low |
| Kluge et al. [8] | 5 | 5 | 2 | 4 | 4 | 20 | Moderate |
| Li et al. [9] | 0 | 5 | 5 | 5 | 5 | 20 | Moderate |
| Luo et al. [10] | 0 | 5 | 5 | 4 | 4 | 18 | Moderate |
| Mair et al. [11] | 0 | 5 | 1 | 3 | 3 | 12 | Low |
| Omogbenigun et al. [12] | 0 | 5 | 2 | 3 | 4 | 14 | Low |
| Upadhaya et al. [13] | 0 | 5 | 4 | 3 | 4 | 16 | Moderate |
| Walsh et al. [14] | 0 | 5 | 5 | 3 | 4 | 17 | Moderate |
| Yang et al. [15] | 0 | 5 | 5 | 4 | 5 | 19 | Moderate |
| Yen et al. [16] | 0 | 5 | 5 | 3 | 2 | 15 | Moderate |
| Espinosa et al. [17] | 0 | 5 | 4 | 4 | 4 | 17 | Moderate |
| Han et al. [18] | 0 | 5 | 5 | 4 | 2 | 16 | Moderate |
| Jlali et al. [19] | 0 | 5 | 4 | 4 | 4 | 17 | Moderate |
| Li et al. [20] | 0 | 5 | 3 | 4 | 4 | 16 | Moderate |
| Marcolla et al. [21] | 5 | 5 | 2 | 3 | 4 | 19 | Moderate |
| Mateo et al. [22] | 0 | 5 | 5 | 4 | 4 | 18 | Moderate |
| Choi et al. [23] | 0 | 5 | 5 | 5 | 4 | 19 | Moderate |
| Chu et al. [24] | 0 | 5 | 5 | 4 | 4 | 18 | Moderate |
| Hancox et al. [25] | 0 | 5 | 2 | 3 | 2 | 12 | Low |
| Kim et al. [26] | 5 | 5 | 2 | 5 | 4 | 21 | High |
| Lan et al. [27] | 0 | 5 | 4 | 4 | 4 | 17 | Moderate |
| Li et al. [29] | 0 | 5 | 3 | 4 | 4 | 16 | Moderate |
| Liu et al. [30] | 0 | 5 | 2 | 4 | 4 | 15 | Moderate |
| Shen et al. [31] | 0 | 5 | 5 | 5 | 4 | 19 | Moderate |
| van Heugten et al. [32] | 0 | 5 | 3 | 3 | 4 | 15 | Moderate |
| Hu et al. [33] | 0 | 5 | 2 | 5 | 4 | 16 | Moderate |
| Manzanilla et al. [34] | 0 | 5 | 1 | 4 | 5 | 15 | Moderate |
| Pan et al. [35] | 0 | 5 | 3 | 3 | 4 | 15 | Moderate |
| Partanen et al. [36] | 0 | 5 | 3 | 4 | 4 | 16 | Moderate |
| Qian et al. [37] | 0 | 5 | 3 | 4 | 4 | 16 | Moderate |
| van der Peet-Schwering et al. [38] | 0 | 5 | 5 | 5 | 5 | 20 | High |
| Van Nevel et al. [39] | 0 | 5 | 1 | 3 | 3 | 12 | Low |
| Walsh et al. [40] | 0 | 5 | 1 | 2 | 3 | 11 | Low |
| Yeh et al. [41] | 0 | 5 | 2 | 4 | 4 | 15 | Moderate |
| Zhao et al. [42] | 0 | 5 | 3 | 4 | 4 | 16 | Moderate |
| Lee et al. [43] | 0 | 5 | 2 | 5 | 5 | 17 | Moderate |
| Superchi et al. [44] | 5 | 5 | 4 | 3 | 4 | 21 | High |
| Waititu et al. [45] | 0 | 5 | 2 | 4 | 4 | 15 | Moderate |
| Wang et al. [46] | 0 | 5 | 3 | 4 | 4 | 16 | Moderate |
| Yi et al. [47] | 0 | 5 | 4 | 4 | 5 | 18 | Moderate |
| Chen et al. [48] | 0 | 5 | 5 | 3 | 4 | 17 | Moderate |
| Clarke et al. [49] | 0 | 5 | 2 | 3 | 4 | 14 | Low |
| Davis et al. [50] | 0 | 5 | 2 | 4 | 4 | 15 | Moderate |
| Fang et al. [51] | 0 | 5 | 2 | 3 | 4 | 14 | Low |
| Jo et al. [52] | 0 | 5 | 3 | 3 | 4 | 15 | Moderate |
| Lan et al. [53] | 0 | 5 | 5 | 3 | 4 | 17 | Moderate |
| Molist et al. [54] | 0 | 5 | 2 | 4 | 4 | 15 | Moderate |
| Omogbenigun et al. [55] | 0 | 5 | 1 | 3 | 3 | 12 | Low |
| Owusu-Asiedu et al. [56] | 0 | 5 | 5 | 3 | 4 | 17 | Moderate |
| Shan et al. [57] | 0 | 5 | 3 | 5 | 5 | 18 | Moderate |
| Taylor et al. [58] | 0 | 5 | 5 | 3 | 4 | 17 | Moderate |
| Vahjen et al. [59] | 0 | 5 | 2 | 3 | 4 | 14 | Low |
| Walk et al. [60] | 0 | 5 | 4 | 3 | 4 | 16 | Moderate |
| Zhang et al. [61] | 0 | 5 | 5 | 4 | 4 | 18 | Moderate |
| Zhang et al. [62] | 0 | 5 | 5 | 3 | 4 | 17 | Moderate |
| Long et al. [63] | 0 | 5 | 5 | 4 | 5 | 19 | Moderate |
| Oliver and Wells [64] | 5 | 5 | 5 | 3 | 4 | 22 | High |
| Oliver et al. [65] | 0 | 5 | 5 | 3 | 4 | 17 | Moderate |
| Hosseindoust et al. [66] | 0 | 5 | 4 | 4 | 4 | 17 | Moderate |
| Kim et al. [67] | 0 | 5 | 5 | 5 | 5 | 20 | High |
| Begum et al. [68] | 0 | 5 | 4 | 4 | 5 | 18 | Moderate |
| Chen et al. [69] | 0 | 5 | 1 | 3 | 4 | 13 | Low |
| Cheng et al. [70] | 0 | 5 | 2 | 4 | 4 | 15 | Moderate |
| Di Giancamillo et al. [71] | 0 | 5 | 1 | 3 | 3 | 12 | Low |
| Ilsley et al. [72] | 0 | 5 | 5 | 4 | 5 | 19 | Moderate |
| Jeong and Kim [73] | 0 | 5 | 5 | 4 | 5 | 19 | Moderate |
| Kang et al. [74] | 5 | 5 | 1 | 4 | 5 | 20 | Moderate |
| Lei, Lee et al. [75] | 0 | 5 | 2 | 4 | 4 | 15 | Moderate |
| Li and Kim [28] | 0 | 5 | 4 | 4 | 4 | 17 | Moderate |
| Manzanilla et al. [76] | 0 | 5 | 5 | 4 | 4 | 18 | Moderate |
| Nowak et al. [77] | 0 | 5 | 1 | 4 | 3 | 13 | Low |
| Schone et al. [78] | 5 | 5 | 2 | 4 | 5 | 21 | High |
| Wang et al. [79] | 0 | 5 | 3 | 4 | 5 | 17 | Moderate |
| Yan and Kim [80] | 0 | 5 | 4 | 4 | 5 | 18 | Moderate |
| Yan et al. [81] | 0 | 5 | 4 | 4 | 5 | 18 | Moderate |
| Yin et al. [82] | 0 | 5 | 2 | 3 | 4 | 14 | Low |
| Cutler et al. [83] | 5 | 5 | 1 | 4 | 4 | 19 | Moderate |
| Tang et al. [84] | 0 | 5 | 1 | 4 | 5 | 15 | Moderate |
| Wu et al. [85] | 0 | 5 | 1 | 4 | 5 | 15 | Moderate |
| Xiao et al. (1)[86] | 0 | 5 | 1 | 4 | 4 | 14 | Low |
| Xiao et al. (2)[87] | 0 | 0 | 1 | 4 | 5 | 10 | Low |
| Yoon et al. [88] | 0 | 0 | 2 | 4 | 5 | 11 | Low |
| Yoon et al. [89] | 0 | 5 | 5 | 4 | 5 | 19 | Moderate |

Within-group differences: Within-group SD/SE not reported-0’

Multiple reports: The same batch of pigs was reported in several articles-0’

Sample size: 150-5’; 100-4’; 70-3’; 30-2’; <30-1’

quality: >20'-high; 15-20’-moderate; <15'-low

**Table S4.** Minimal inhibitory concentration table (μg/mL)

| **Type** | **Bacteria** | **Antimicrobial peptides** | | | | | | | | | | |
| --- | --- | --- | --- | --- | --- | --- | --- | --- | --- | --- | --- | --- |
|  |  | **PMAP-23** [90] | **PG-1** [91] | **PMAP-37** [92] | **Cecropin P1** [93] | **Ranalexin-1G** [94] | **Px-cec1** [95] | **CecropinXJ** [96] | **pxCECA1** [97] | **Cathelicidin-AL** [98] | **Lf-CATH1** [99] | **Cathelicidin-PY** [100] |
| Gram positive | *Staphylococcus aureus* | 12 | 25 | 606 | 1637 | 38 | 8 | 7 | 8 | 6 | 4 | 9 |
|  | *Bacillus subtilis* |  |  |  |  |  | 10 |  | 8 | 100 |  | >100 |
|  | *Bacillus megaterium* | 6 |  | 76 | 6 |  |  |  |  |  |  |  |
| Gram negative | *Escherichia coli* | 6 | 25 | 19 | 1 | 19 | 0.40 | 6 | 4 | 25 | 8 | 5 |
|  | *Salmonella Typhimurium* | 24 |  | 76 | 3 |  |  |  |  |  |  |  |
|  | *Pseudomonas aeruginosa* | 47 |  | 38 | 20 |  |  |  | 8 | 6 | 32 | 19 |
|  | *Proteus vulgaris* |  |  |  | 19 |  |  |  |  |  | >105 |  |
| **Type** | **Bacteria** | **Plant extracts** | | | | | | | | | | |
|  |  | ***Cinnamomum zeylanicum* essential oil** [101] | ***Tetradenia riparia* essential oil** [102] | ***Combretum adenogonium* Steud. Ex A. Rich** [103] | ***Mitracarpus frigidus* essential oil** [104] | **Oregano [105, 106]** | ***Angophora leiocarpa* seed lectin** [107] | ***Pimelea longiflora***  **essential oil** [108] | ***Psidium guajava*** [108] | **Thymol [106, 108-113]** | ***Satureja horvatii* essential oil** [114] | **Tea polyphenols** [115] |
| Gram positive | *Staphylococcus aureus* | 560 | 16 | 2500 | 250 | 900; 725 | 181 | 128 | 1250 | 365; 512 | 150 | 156 |
|  | *Listeria monocytogenes* | 560 |  |  |  |  |  |  |  | 250; 188 | 570 | 156 |
|  | *Bacillus subtilis* |  | 7 |  |  | 2250 | 45 | 128 |  | 128 |  | 1250 |
|  | *Staphylococcus epidermidis* |  |  |  |  | 670 |  |  | 39 | 1300 |  |  |
|  | *Enterococcus faecalis* | 1120 | 63 |  | 1000 |  | 90 |  | 194 |  |  |  |
|  | *Bacillus cereus* | 560 |  | 1250 | 250 |  | 45 |  |  |  |  |  |
|  | *Streptococcus pyogenes* | 560 |  |  | 500 |  | 181 |  |  |  |  |  |
| Gram negative | *Escherichia coli* | 1120 | 125±0 | 5000 | 500 | 900 | 181 | 256 | ˃5000 | 94, 1024 | 30±20 | 313 |
|  | *Salmonella Typhimurium* |  |  | 1250 | 1000 | 350 |  | 256 |  | 1024; 950 | 30 |  |
|  | *Klebsiella pneumoniae* | 140 | 125 | 5000 | 500 |  | 45 |  | 5000 |  |  |  |
|  | *Salmonella Enteritidis* |  | 500 |  |  |  | 181 |  | 5000 | 640; 188 |  | 625 |
|  | *Pseudomonas aeruginosa* | 280 | 125 | 1250 | 250 |  | 181 | 512 |  | 1024 |  | 313 |
|  | *Shigella flexneri* |  |  | >10,000 |  |  |  |  | 625 |  |  |  |
| **Type** | **Bacteria** | **Plant extracts** | | | | | | | | | | |
|  |  | ***Lippia graveolens* essential oil** [108] | **Carvacrol [108, 110, 112]** | **Eugenol [110, 112]** | **Cinnamon oil** [111] | **Ethanol extract** [116] | **Kelantan** [117] | **Johor** [117] | **Pahang** [117] | **Rosemary** [105] | **Lauric arginate** [118] |  |
| Gram positive | *Staphylococcus aureus* | 512 | 128 |  | 325 | 1000 | 40 | 60 | 30 | 725 |  |  |
|  | *Listeria monocytogenes* |  |  | 750; 1500 | 750 |  | 40 |  | 50 |  | 12 |  |
|  | *Bacillus subtilis* | 512 | 128 |  |  |  | 80 | >100 | >100 |  |  |  |
| Gram negative | *Escherichia coli* | 512 | 128 | 750; 1000 | 750 | >1000 | >100 |  | >100 | 4400 | 12 |  |
|  | *Salmonella Typhimurium* | 512 | 320; 128 | 640 | 350 | 250 | 50 | >100 | >100 | 350 |  |  |
|  | *Klebsiella pneumoniae* |  |  |  |  | 250 |  |  |  |  |  |  |
|  | *Salmonella Enteritidis* |  | 640 | 640; 750 | 750 |  |  |  |  |  | 24 |  |
|  | *Pseudomonas aeruginosa* |  | 128 |  |  |  | 60 |  | 80 |  |  |  |
| **Type** | **Bacteria** | **Antibiotics** | | | | | | | | | | |
|  |  | **Penicillin** [116] | **Amoxicillin** [107] | **Chloramphenicol** [104] | **Gentamycin** [119] | **Amikacin** [112] | **Oxytetracycline** [120] | **Norfloxacin** [116] | **Cephalothin** [119] | **Streptomycin** [114] | **Cycloheximide** [114] |  |
| Gram positive | *Staphylococcus aureus* |  | 8 | 63 | 1 | 2 | 20 | 500 | 1 | 50 | 100 |  |
|  | *Streptococcus agalactiae* | 100 |  |  |  |  |  | 30 |  |  |  |  |
|  | *Listeria monocytogenes* |  |  |  |  |  | 20 |  |  | 50 | 100 |  |
|  | *Enterococcus faecalis* |  | 4 | 31 |  |  |  |  | 8 |  |  |  |
|  | *Bacillus cereus* |  | 16 | 4 | 1 |  |  |  |  |  |  |  |
|  | *Streptococcus pyogenes* |  | 8 | 16 |  |  |  |  |  |  |  |  |
| Gram negative | *Escherichia coli* | 100 | 16 | 16 | 2 | 2 | 10 | 250 | 10±0 | 200±10 | 400±10 |  |
|  | *Salmonella typhimurium* | 100 |  | 1 | 6 |  | 10 |  |  | 100±20 | 200±20 |  |
|  | *Klebsiella pneumoniae* | 100 | 31 | 1 | 3 | 0.25 |  |  |  |  |  |  |
|  | *Salmonella enteritidis* |  | 16 |  |  | 0.13 |  |  |  |  |  |  |
|  | *Pseudomonas aeruginosa* |  | 8 | 16 | 2 | 1 | 10 |  |  |  |  |  |
| **Type** | **Bacteria** | **Organic acids** | | | | | | | | **Microelements** | | |
|  |  | **Diterpene ent-dihydrotumanoic acid (DTA)** [121] | **Oleanolic acid** [120] | **Lactobionic acid** [113] | **Lauric acid** [122] | **Lactic acid** [123]**]** | **Cinnamic acid** [123] | **Propionic acid** [123] | **Capric acid** [121] | **Gram negative Bacteria** | **Silver nanoparticles** [124] | **Zinc oxide nanoparticles** [124] |
| Gram positive | *Staphylococcus aureus* | 250-500 | 35 |  | 50 |  |  |  | 100 | *Aeromonas hydrophila* | 17 | 16 |
|  | *Listeria monocytogenes* |  | 30 | 10,000 |  |  |  |  |  | *Aeromonas salmonicida* | 17 | 16 |
|  | *Bacillus subtilis* | 125 | 30 |  |  |  |  |  |  | *Yersinia ruckeri* | 17 | 32 |
| Gram negative | *Escherichia coli* | 500 | 50 | 10,000 |  |  |  |  |  | *Aphanomyces invadans* | 17 | 32 |
|  | *Salmonella Typhimurium* | 500 | 100 |  |  | 3000 | 2000 | 1000 |  |  |  |  |
|  | *Klebsiella pneumoniae* | 500 |  |  |  |  |  |  |  |  |  |  |
|  | *Pseudomonas aeruginosa* | 500 | 40 |  |  |  |  |  |  |  |  |  |
| **Type** | **Item** | Gram positive bacteria | | | | Gram negative bacteria | | |  |  |  | |
|  |  | *Staphylococcus aureus* | *Brochothrix thermosphacta* | *Listeria monocytogenes* | *Bacillus subtilis* | *Escherichia coli* | *Salmonella Typhimurium* | *Klebsiella pneumoniae* |  |  |  |  |
| **Oligosaccharides** | **Chitosan** [115] | 938 | 469 | 234 | 938 | 469 | 469 | 469 |  |  |  |  |
| **Type** | **Item** | Gram positive bacteria | | | | |  | Gram negative bacteria | | | | |
| **Lysozyme** |  | *Brochothrix thermosphacta* | *Pediococcus acidilactici* | *Leuconostoc mesenteroides* | *Staphylococcus aureus* | *Staphylococcus pasteuri* | *Vibrio parahaemolyticus* | *Enterobacter aerogenes* | *Enterobacter cloacae* | *Pseudomonas putida* | *Proteus mirabilis* | *Vibrio azureus* |
|  | **Lysozyme** [125] | 4 | >500 | 500 | 938 |  |  |  |  |  |  |  |
|  | **rMGgLYZ1** [126] |  |  |  |  | 20-41 | 20-41 | 40-79 | 41 -82 | 20-41 | 41 -82 | 41 -82 |
|  | **rMGgLYZ2** [126] |  |  |  |  | 5-50 | 5-50 | 5-50 | 5-50 | 2-25 | 5-50 | 5-50 |

**Table S5.** Meta-analyses and subgroup analyses

| **Outcomes** | **Growth stages** | **Type of feed additives** | **NO. trials** | **Pooled estimate (95% CI)** | ***P*** | ***P*_heterogeneity_** | ***I*^2^** | **Egger’s test** |
| --- | --- | --- | --- | --- | --- | --- | --- | --- |
| ADG | Weaned piglets | Oligosaccharide | 4 | SMD 1.311(0.811 to 1.81) | <0.001 | <0.001 | 84.20% |  |
| ADG | Weaned piglets | Nucleotide | 3 | SMD 1.837(-1.187 to 4.861) | 0.234 | <0.001 | 98.30% |  |
| ADG | Weaned piglets | Antimicrobial peptides | 11 | SMD 3.071(2.604 to 3.538) | <0.001 | 0.383 | 0.00% |  |
| ADG | Weaned piglets | Amylase | 24 | SMD 0.818(0.54 to 1.096) | <0.001 | <0.001 | 84.90% |  |
| ADG | Weaned piglets | Lysozyme | 6 | SMD 1.334(0.655 to 2.013) | <0.001 | <0.001 | 91.90% |  |
| ADG | Weaned piglets | Bacteriophages | 3 | SMD 2.433(1.578 to 3.289) | <0.001 | <0.001 | 87.60% |  |
| ADG | Weaned piglets | Organic acid | 24 | SMD 1.034(0.566 to 1.502) | <0.001 | <0.001 | 93.40% |  |
| ADG | Weaned piglets | Microelement | 10 | SMD 1.139(0.436 to 1.842) | 0.002 | <0.001 | 93.10% |  |
| ADG | Weaned piglets | Probiotics | 16 | SMD 1.739(1.187 to 2.292) | <0.001 | <0.001 | 91.30% |  |
| ADG | Weaned piglets | Plant | 4 | SMD 0.665(0.298 to 1.032) | <0.001 | 0.137 | 45.70% |  |
| ADG | Weaned piglets | Plant extract | 18 | SMD 1.018(0.283 to 1.753) | 0.007 | <0.001 | 96.20% |  |
| ADG | Weaned piglets | Overall | 123 | SMD 1.207(0.999 to 1.415) | <0.001 | <0.001 | 93.80% | 0.001 |
| ADG | Growing pigs | Amylase | 10 | SMD 1.222(0.604 to 1.84) | <0.001 | <0.001 | 96.30% |  |
| ADG | Growing pigs | Organic acid | 6 | SMD 0.71(-0.014 to 1.433) | 0.054 | <0.001 | 85.70% |  |
| ADG | Growing pigs | Microelement | 6 | SMD -0.268(-0.553 to 0.018) | 0.066 | 0.614 | 0.00% |  |
| ADG | Growing pigs | Probiotics | 4 | SMD 2.946(1.103 to 4.789) | 0.002 | <0.001 | 95.00% |  |
| ADG | Growing pigs | Plant | 6 | SMD 1.603(0.664 to 2.541) | 0.001 | <0.001 | 91.70% |  |
| ADG | Growing pigs | Plant extract | 4 | SMD 1.948(1.014 to 2.882) | <0.001 | <0.001 | 86.50% |  |
| ADG | Growing pigs | Overall | 36 | SMD 1.223(0.85 to 1.595) | <0.001 | <0.001 | 94.30% | 0.002 |
| ADG | Finishing pigs | Organic acid | 4 | SMD 1.179(0.877 to 1.481) | <0.001 | 0.33 | 12.60% |  |
| ADG | Finishing pigs | Microelement | 5 | SMD -0.654(-1.144 to -0.165) | 0.009 | 0.001 | 77.60% |  |
| ADG | Finishing pigs | Probiotics | 2 | SMD 0.718(0.36 to 1.076) | <0.001 | 0.43 | 0.00% |  |
| ADG | Finishing pigs | Plant | 3 | SMD 1.192(0.511 to 1.873) | 0.001 | 0.051 | 66.40% |  |
| ADG | Finishing pigs | Plant extract | 4 | SMD 0.346(-0.473 to 1.164) | 0.408 | <0.001 | 84.10% |  |
| ADG | Finishing pigs | Overall | 18 | SMD 0.424(0.004 to 0.843) | 0.048 | <0.001 | 89.80% | 0.909 |
| ADFI | Weaned piglets | Oligosaccharide | 4 | SMD 0.73(0.246 to 1.215) | 0.003 | <0.001 | 85.20% |  |
| ADFI | Weaned piglets | Nucleotide | 3 | SMD -0.523(-1.904 to 0.858) | 0.458 | <0.001 | 94.20% |  |
| ADFI | Weaned piglets | Antimicrobial peptides | 11 | SMD 1.916(0.728 to 3.104) | 0.002 | 0.002 | 89.30% |  |
| ADFI | Weaned piglets | Amylase | 24 | SMD -0.048(-0.345 to 0.248) | 0.749 | <0.001 | 87.60% |  |
| ADFI | Weaned piglets | Lysozyme | 4 | SMD 0.366(0.11 to 0.621) | 0.005 | 0.825 | 0.00% |  |
| ADFI | Weaned piglets | Bacteriophages | 3 | SMD 0.499(0.252 to 0.746) | <0.001 | 0.317 | 13.00% |  |
| ADFI | Weaned piglets | Organic acid | 19 | SMD 0.682(0.255 to 1.109) | 0.002 | <0.001 | 91.10% |  |
| ADFI | Weaned piglets | Microelement | 8 | SMD 0.961(0.281 to 1.64) | 0.006 | <0.001 | 91.40% |  |
| ADFI | Weaned piglets | Probiotics | 16 | SMD 1.065(0.266 to 1.865) | 0.009 | <0.001 | 96.20% |  |
| ADFI | Weaned piglets | Plant | 4 | SMD 0.405(-0.344 to 1.155) | 0.289 | <0.001 | 86.70% |  |
| ADFI | Weaned piglets | Plant extract | 18 | SMD 0.431(-0.015 to 0.877) | 0.058 | <0.001 | 91.40% |  |
| ADFI | Weaned piglets | Overall | 114 | SMD 0.515(0.327 to 0.703) | <0.001 | <0.001 | 92.60% | 0.005 |
| ADFI | Growing pigs | Amylase | 11 | SMD -0.122(-0.381 to 0.137) | 0.356 | <0.001 | 82.40% |  |
| ADFI | Growing pigs | Organic acid | 2 | SMD 3.434(2.296 to 4.572) | <0.001 | 0.621 | 0.00% |  |
| ADFI | Growing pigs | Microelement | 6 | SMD -0.325(-0.66 to 0.01) | 0.057 | 0.238 | 26.20% |  |
| ADFI | Growing pigs | Probiotics | 4 | SMD 0.471(-0.096 to 1.039) | 0.103 | 0.008 | 74.50% |  |
| ADFI | Growing pigs | Plant | 6 | SMD -0.269(-1.047 to 0.509) | 0.498 | <0.001 | 90.50% |  |
| ADFI | Growing pigs | Plant extract | 4 | SMD 0.588(-0.333 to 1.51) | 0.211 | <0.001 | 89.80% |  |
| ADFI | Growing pigs | Overall | 33 | SMD 0.07(-0.155 to 0.296) | 0.543 | <0.001 | 85.30% | 0.278 |
| ADFI | Finishing pigs | Microelement | 5 | SMD 0.313(-0.107 to 0.733) | 0.144 | 0.008 | 71.00% |  |
| ADFI | Finishing pigs | Probiotics | 2 | SMD -1.241(-2.184 to -0.298) | 0.01 | 0.014 | 83.40% |  |
| ADFI | Finishing pigs | Plant | 3 | SMD -0.265(-0.78 to 0.25) | 0.313 | 0.133 | 50.40% |  |
| ADFI | Finishing pigs | Plant extract | 4 | SMD -0.405(-0.831 to 0.02) | 0.062 | 0.141 | 45.10% |  |
| ADFI | Finishing pigs | Overall | 14 | SMD -0.232(-0.599 to 0.135) | 0.216 | <0.001 | 83.40% | 0.431 |
| GF | Weaned piglets | Oligosaccharide | 4 | SMD 1.021(0.849 to 1.192) | <0.001 | 0.962 | 0.00% |  |
| GF | Weaned piglets | Nucleotide | 3 | SMD 0.809(-0.271 to 1.889) | 0.142 | <0.001 | 90.70% |  |
| GF | Weaned piglets | Antimicrobial peptides | 11 | SMD 2.252(1.849 to 2.656) | <0.001 | 0.732 | 0.00% |  |
| GF | Weaned piglets | Amylase | 22 | SMD 1.144(0.793 to 1.496) | <0.001 | <0.001 | 89.20% |  |
| GF | Weaned piglets | Lysozyme | 6 | SMD 0.771(0.218 to 1.323) | 0.006 | <0.001 | 89.20% |  |
| GF | Weaned piglets | Bacteriophages | 3 | SMD 2.595(1.67 to 3.52) | <0.001 | <0.001 | 88.70% |  |
| GF | Weaned piglets | Organic acid | 24 | SMD 1.371(0.898 to 1.845) | <0.001 | <0.001 | 93.30% |  |
| GF | Weaned piglets | Microelement | 11 | SMD 0.548(0.122 to 0.974) | 0.012 | <0.001 | 85.40% |  |
| GF | Weaned piglets | Probiotics | 14 | SMD 0.872(0.207 to 1.536) | 0.01 | <0.001 | 93.80% |  |
| GF | Weaned piglets | Plant | 3 | SMD 0.455(0.041 to 0.869) | 0.031 | 0.098 | 56.90% |  |
| GF | Weaned piglets | Plant extract | 18 | SMD 0.417(-0.139 to 0.972) | 0.142 | <0.001 | 94.30% |  |
| GF | Weaned piglets | Overall | 119 | SMD 0.979(0.79 to 1.168) | <0.001 | <0.001 | 92.60% | 0.033 |
| GF | Growing pigs | Amylase | 7 | SMD -0.056(-0.439 to 0.327) | 0.774 | <0.001 | 88.50% |  |
| GF | Growing pigs | Organic acid | 4 | SMD 0.011(-0.249 to 0.27) | 0.935 | 0.493 | 0.00% |  |
| GF | Growing pigs | Microelement | 6 | SMD -0.015(-0.3 to 0.269) | 0.915 | 0.686 | 0.00% |  |
| GF | Growing pigs | Probiotics | 5 | SMD 0.967(-0.454 to 2.388) | 0.182 | <0.001 | 96.30% |  |
| GF | Growing pigs | Plant | 6 | SMD 1.576(0.938 to 2.213) | <0.001 | <0.001 | 82.60% |  |
| GF | Growing pigs | Plant extract | 4 | SMD 0.572(-0.269 to 1.414) | 0.183 | <0.001 | 87.80% |  |
| GF | Growing pigs | Overall | 32 | SMD 0.491(0.177 to 0.806) | 0.002 | <0.001 | 92.10% | <0.001 |
| GF | Finishing pigs | Organic acid | 4 | SMD 0.209(-0.051 to 0.468) | 0.116 | 0.803 | 0.00% |  |
| GF | Finishing pigs | Microelement | 5 | SMD -0.801(-1.243 to -0.359) | <0.001 | 0.007 | 71.90% |  |
| GF | Finishing pigs | Probiotics | 2 | SMD 3.066(2.55 to 3.582) | <0.001 | 0.466 | 0.00% |  |
| GF | Finishing pigs | Plant | 3 | SMD 0.338(-0.023 to 0.699) | 0.067 | 0.667 | 0.00% |  |
| GF | Finishing pigs | Plant extract | 3 | SMD 0.769(0.259 to 1.28) | 0.003 | 0.724 | 0.00% |  |
| GF | Finishing pigs | Overall | 17 | SMD 0.336(-0.18 to 0.853) | 0.201 | <0.001 | 92.20% | 0.042 |
| Duodenum V/C | Weaned piglets | Oligosaccharide | 4 | SMD 0.389(-1.222 to 2) | 0.636 | <0.001 | 89.80% |  |
| Duodenum V/C | Weaned piglets | Antimicrobial peptides | 4 | SMD 2.144(1.64 to 2.648) | <0.001 | <0.001 | 90.1% |  |
| Duodenum V/C | Weaned piglets | Lysozyme | 2 | SMD 2.478(0.955 to 4.002) | 0.001 | 0.002 | 89.60% |  |
| Duodenum V/C | Weaned piglets | Bacteriophages | 3 | SMD 0.227(-0.206 to 0.66) | 0.305 | 0.027 | 72.20% |  |
| Duodenum V/C | Weaned piglets | Organic acid | 3 | SMD 2.475(1.562 to 3.388) | <0.001 | <0.001 | 88.50% |  |
| Duodenum V/C | Weaned piglets | Microelement | 4 | SMD 4.093(2.064 to 6.121) | <0.001 | <0.001 | 95.60% |  |
| Duodenum V/C | Weaned piglets | Probiotics | 1 | SMD 0.69(0.286 to 1.093) | 0.001 | NA | NA |  |
| Duodenum V/C | Weaned piglets | Plant extract | 1 | SMD -1.977(-3.066 to -0.888) | <0.001 | NA | NA |  |
| Duodenum V/C | Weaned piglets | Overall | 22 | SMD 1.667(1.012 to 2.322) | <0.001 | <0.001 | 95.50% | 0.115 |
| Duodenum V/C | Growing pigs | Organic acid | 2 | SMD 1.794(0.417 to 3.171) | 0.011 | 0.111 | 60.70% |  |
| Duodenum V/C | Growing pigs | Overall | 2 | SMD 1.794(0.417 to 3.171) | 0.011 | 0.111 | 60.70% | NA |
| Jejunum V/C | Weaned piglets | Oligosaccharide | 8 | SMD 0.367(-0.236 to 0.97) | 0.233 | <0.001 | 87.60% |  |
| Jejunum V/C | Weaned piglets | Nucleotide | 1 | SMD 1.318(-0.256 to 2.892) | 0.101 | NA | NA |  |
| Jejunum V/C | Weaned piglets | Antimicrobial peptides | 6 | SMD 3.069(-0.356 to 6.494) | 0.079 | <0.001 | 98.00% |  |
| Jejunum V/C | Weaned piglets | Lysozyme | 2 | SMD 1.9(0.676 to 3.125) | 0.002 | 0.006 | 87.00% |  |
| Jejunum V/C | Weaned piglets | Bacteriophages | 3 | SMD 0.314(0.086 to 0.542) | 0.007 | 0.409 | 0.00% |  |
| Jejunum V/C | Weaned piglets | Organic acid | 7 | SMD 0.314(-0.635 to 1.263) | 0.517 | <0.001 | 93.00% |  |
| Jejunum V/C | Weaned piglets | Microelement | 4 | SMD 6.34(3.188 to 9.491) | <0.001 | <0.001 | 97.10% |  |
| Jejunum V/C | Weaned piglets | Probiotics | 1 | SMD 0.574(0.174 to 0.974) | 0.005 | NA | NA |  |
| Jejunum V/C | Weaned piglets | Plant extract | 2 | SMD 1.729(-1.542 to 5) | 0.3 | <0.001 | 93.00% |  |
| Jejunum V/C | Weaned piglets | Overall | 34 | SMD 1.44(0.923 to 1.957) | <0.001 | <0.001 | 95.70% | 0.001 |
| Jejunum V/C | Growing pigs | Organic acid | 2 | SMD 1.468(-1.308 to 4.243) | 0.3 | 0.002 | 89.90% |  |
| Jejunum V/C | Growing pigs | Plant extract | 1 | SMD 1.8(0.839 to 2.761) | <0.001 | NA | NA |  |
| Jejunum V/C | Growing pigs | Overall | 3 | SMD 1.546(0.003 to 3.09) | 0.05 | 0.003 | 82.70% | 0.535 |
| Jejunum V/C | Finishing pigs | Plant extract | 1 | SMD 1.8(0.839 to 2.761) | <0.001 | NA | NA |  |
| Jejunum V/C | Finishing pigs | Overall | 1 | SMD 1.8(0.839 to 2.761) | <0.001 | NA | NA | NA |
| Ileum V/C | Weaned piglets | Oligosaccharide | 4 | SMD -1.37(-1.879 to -0.862) | <0.001 | 0.366 | 5.40% |  |
| Ileum V/C | Weaned piglets | Nucleotide | 2 | SMD 0.87(-0.26 to 2) | 0.131 | 0.128 | 56.90% |  |
| Ileum V/C | Weaned piglets | Antimicrobial peptides | 5 | SMD 3.134(2.534 to 3.735) | <0.001 | <0.001 | 94.4% |  |
| Ileum V/C | Weaned piglets | Lysozyme | 2 | SMD 0.233(-0.127 to 0.592) | 0.204 | 0.585 | 0.00% |  |
| Ileum V/C | Weaned piglets | Bacteriophages | 3 | SMD 0.825(0.018 to 1.632) | 0.045 | <0.001 | 91.20% |  |
| Ileum V/C | Weaned piglets | Organic acid | 5 | SMD 0.268(-0.232 to 0.769) | 0.293 | 0.005 | 73.10% |  |
| Ileum V/C | Weaned piglets | Microelement | 4 | SMD 2.646(1.542 to 3.75) | <0.001 | <0.001 | 90.00% |  |
| Ileum V/C | Weaned piglets | Probiotics | 1 | SMD 0.056(-0.336 to 0.448) | 0.779 | NA | NA |  |
| Ileum V/C | Weaned piglets | Plant extract | 2 | SMD 1.866(0.734 to 2.998) | 0.001 | 0.161 | 49.10% |  |
| Ileum V/C | Weaned piglets | Overall | 28 | SMD 0.768(0.301 to 1.236) | 0.001 | <0.001 | 93.00% | 0.701 |
| Ileum V/C | Growing pigs | Organic acid | 2 | SMD 3.126(1.312 to 4.94) | 0.001 | 0.104 | 62.30% |  |
| Ileum V/C | Growing pigs | Plant extract | 1 | SMD 1.2(0.325 to 2.075) | 0.007 | NA | NA |  |
| Ileum V/C | Growing pigs | Overall | 3 | SMD 2.392(0.828 to 3.957) | 0.003 | 0.012 | 77.20% | 0.056 |
| Ileum V/C | Finishing pigs | Plant extract | 1 | SMD 1.2(0.325 to 2.075) | 0.007 | NA | NA |  |
| Ileum V/C | Finishing pigs | Overall | 1 | SMD 1.2(0.325 to 2.075) | 0.007 | NA | NA | NA |
| IgA | Weaned piglets | Nucleotide | 2 | SMD 0.855(-0.685 to 2.395) | 0.276 | 0.089 | 65.50% |  |
| IgA | Weaned piglets | Amylase | 1 | SMD 2.309(1.652 to 2.967) | <0.001 | NA | NA |  |
| IgA | Weaned piglets | Bacteriophages | 1 | SMD 0.233(-0.161 to 0.626) | 0.247 | NA | NA |  |
| IgA | Weaned piglets | Organic acid | 2 | SMD 0.637(0.009 to 1.264) | 0.047 | 0.322 | 0.00% |  |
| IgA | Weaned piglets | Plant extract | 3 | SMD 0.406(-0.631 to 1.443) | 0.443 | <0.001 | 95.70% |  |
| IgA | Weaned piglets | Overall | 9 | SMD 0.755(0.178 to 1.333) | 0.01 | <0.001 | 90.10% | 0.682 |
| IgA | Finishing pigs | Plant extract | 2 | SMD 1.392(1.027 to 1.757) | <0.001 | 0.65 | 0.00% |  |
| IgA | Finishing pigs | Overall | 2 | SMD 1.392(1.027 to 1.757) | <0.001 | 0.65 | 0.00% | NA |
| IgM | Weaned piglets | Nucleotide | 2 | SMD 0.39(-0.225 to 1.004) | 0.214 | 0.449 | 0.00% |  |
| IgM | Weaned piglets | Amylase | 1 | SMD 1.443(0.873 to 2.014) | <0.001 | NA | NA |  |
| IgM | Weaned piglets | Bacteriophages | 1 | SMD -2.069(-2.556 to -1.582) | <0.001 | NA | NA |  |
| IgM | Weaned piglets | Organic acid | 2 | SMD 0.14(-0.47 to 0.749) | 0.653 | 0.342 | 0.00% |  |
| IgM | Weaned piglets | Plant extract | 1 | SMD 3.175(2.758 to 3.593) | <0.001 | NA | NA |  |
| IgM | Weaned piglets | Overall | 7 | SMD 0.515(-1.13 to 2.16) | 0.54 | <0.001 | 97.80% | 0.632 |
| IgM | Finishing pigs | Plant extract | 2 | SMD 0.289(-0.04 to 0.617) | 0.085 | 1 | 0.00% |  |
| IgM | Finishing pigs | Overall | 2 | SMD 0.289(-0.04 to 0.617) | 0.085 | 1 | 0.00% | NA |
| IgG | Weaned piglets | Nucleotide | 2 | SMD 0.073(-0.535 to 0.682) | 0.814 | 0.386 | 0.00% |  |
| IgG | Weaned piglets | Amylase | 1 | SMD 2.047(1.419 to 2.675) | <0.001 | NA | NA |  |
| IgG | Weaned piglets | Bacteriophages | 1 | SMD -0.5(-0.898 to -0.102) | 0.014 | NA | NA |  |
| IgG | Weaned piglets | Organic acid | 2 | SMD -0.314(-0.926 to 0.299) | 0.315 | 0.34 | 0.00% |  |
| IgG | Weaned piglets | Microelement | 3 | SMD 0.818(0.423 to 1.213) | <0.001 | 0.264 | 24.80% |  |
| IgG | Weaned piglets | Plant extract | 6 | SMD 1.225(-0.538 to 2.989) | 0.173 | <0.001 | 98.70% |  |
| IgG | Weaned piglets | Overall | 15 | SMD 0.733(-0.096 to 1.561) | 0.083 | <0.001 | 96.80% | 0.376 |
| IgG | Growing pigs | Plant | 3 | SMD 0.347(-0.014 to 0.708) | 0.06 | 0.792 | 0.00% |  |
| IgG | Growing pigs | Plant extract | 2 | SMD 2.686(1.485 to 3.888) | <0.001 | 0.01 | 84.90% |  |
| IgG | Growing pigs | Overall | 5 | SMD 1.284(0.154 to 2.414) | 0.026 | <0.001 | 93.70% | 0.397 |
| IgG | Finishing pigs | Plant | 3 | SMD 0.471(0.108 to 0.834) | 0.011 | 0.932 | 0.00% |  |
| IgG | Finishing pigs | Plant extract | 2 | SMD 4.238(1.315 to 7.16) | 0.004 | <0.001 | 95.50% |  |
| IgG | Finishing pigs | Overall | 5 | SMD 1.948(0.351 to 3.545) | 0.017 | <0.001 | 96.30% | 0.065 |
| Lymphocyte | Weaned piglets | Oligosaccharide | 2 | SMD -0.073(-0.747 to 0.601) | 0.832 | <0.001 | 92.80% |  |
| Lymphocyte | Weaned piglets | Amylase | 2 | SMD 2.472(1.923 to 3.022) | <0.001 | 0.445 | 0.00% |  |
| Lymphocyte | Weaned piglets | Lysozyme | 4 | SMD -0.082(-0.366 to 0.202) | 0.573 | 0.29 | 20.00% |  |
| Lymphocyte | Weaned piglets | Plant | 3 | SMD 1.037(0.231 to 1.843) | 0.012 | <0.001 | 87.10% |  |
| Lymphocyte | Weaned piglets | Plant extract | 2 | SMD 1.383(0.559 to 2.207) | 0.001 | 0.012 | 84.00% |  |
| Lymphocyte | Weaned piglets | Overall | 13 | SMD 0.767(0.286 to 1.248) | 0.002 | <0.001 | 93.40% | 0.018 |
| Lymphocyte | Growing pigs | Plant | 5 | SMD 0.432(0.176 to 0.689) | 0.001 | 0.676 | 0.00% |  |
| Lymphocyte | Growing pigs | Plant extract | 2 | SMD 2.506(1.834 to 3.177) | <0.001 | 0.134 | 55.50% |  |
| Lymphocyte | Growing pigs | Overall | 7 | SMD 1.026(0.287 to 1.766) | 0.007 | <0.001 | 90.90% | 0.259 |
| Lymphocyte | Finishing pigs | Plant | 3 | SMD 0.87(0.495 to 1.246) | <0.001 | 0.9 | 0.00% |  |
| Lymphocyte | Finishing pigs | Overall | 3 | SMD 0.87(0.495 to 1.246) | <0.001 | 0.9 | 0.00% | 0.017 |
| Diarrhoea rate | Weaned piglets | Oligosaccharide | 2 | SMD -1.027(-2.579 to 0.524) | 0.194 | <0.001 | 92.80% |  |
| Diarrhoea rate | Weaned piglets | Amylase | 1 | SMD -2.469(-3.145 to -1.792) | <0.001 | NA | NA |  |
| Diarrhoea rate | Weaned piglets | Microelement | 3 | SMD -1.38(-2.234 to -0.527) | 0.002 | 0.001 | 84.90% |  |
| Diarrhoea rate | Weaned piglets | Plant extract | 2 | SMD -1.803(-2.085 to -1.52) | <0.001 | 0.772 | 0.00% |  |
| Diarrhoea rate | Weaned piglets | Overall | 8 | SMD -1.529(-2.004 to -1.053) | <0.001 | <0.001 | 84.20% | 0.77 |
| Diarrhoea index | Weaned piglets | Amylase | 6 | SMD -2.217(-2.695 to -1.739) | <0.001 | 0.021 | 65.50% |  |
| Diarrhoea index | Weaned piglets | Organic acid | 8 | SMD -1.715(-2.798 to -0.632) | 0.002 | <0.001 | 95.30% |  |
| Diarrhoea index | Weaned piglets | Probiotics | 2 | SMD -1.988(-2.7 to -1.276) | <0.001 | NA | NA |  |
| Diarrhoea index | Weaned piglets | Overall | 14 | SMD -1.957(-2.556 to -1.358) | <0.001 | <0.001 | 91.60% | 0.604 |

**References**

1. Biagi G, Piva A, Moschini M, Vezzali E, Roth FX. Effect of gluconic acid on piglet growth performance, intestinal microflora, and intestinal wall morphology. J Anim Sci. 2006;84:370-8.

2. Biagi G, Piva A, Moschini M, Vezzali E, Roth FX. Performance, intestinal microflora, and wall morphology of weanling pigs fed sodium butyrate. J Anim Sci. 2007;85:1184-91.

3. Bosi P, Merialdi G, Scandurra S, Messori S, Bardasi L, Nisi I, et al. Feed supplemented with 3 different antibiotics improved food intake and decreased the activation of the humoral immune response in healthy weaned pigs but had differing effects on intestinal microbiota. J Anim Sci. 2011;89:4043-53.

4. Chen JL, Zheng P, Zhang C, Yu B, He J, Yu J, et al. Benzoic acid beneficially affects growth performance of weaned pigs which was associated with changes in gut bacterial populations, morphology indices and growth factor gene expression. J Anim Physiol Anim Nutr (Berl). 2017;101:1137-46.

5. Chu GM, Jung CK, Kim HY, Ha JH, Kim JH, Jung MS, et al. Effects of bamboo charcoal and bamboo vinegar as antibiotic alternatives on growth performance, immune responses and fecal microflora population in fattening pigs. Anim Sci J. 2013;84:113-20.

6. Fang CL, Sun H, Wu J, Niu HH, Feng J. Effects of sodium butyrate on growth performance, haematological and immunological characteristics of weanling piglets. J Anim Physiol Anim Nutr (Berl). 2014;98:680-5.

7. Kaewtapee C, Krutthai N, Poosuwan K, Poeikhampha T, Koonawootrittriron S, Bunchasak C. Effects of adding liquid DL-methionine hydroxy analogue-free acid to drinking water on growth performance and small intestinal morphology of nursery pigs. J Anim Physiol Anim Nutr (Berl). 2010;94:395-404.

8. Kluge H, Broz J, Eder K. Effect of benzoic acid on growth performance, nutrient digestibility, nitrogen balance, gastrointestinal microflora and parameters of microbial metabolism in piglets. J Anim Physiol Anim Nutr (Berl). 2006;90:316-24.

9. Li S, Zheng J, Deng K, Chen L, Zhao XL, Jiang X, et al. Supplementation with organic acids showing different effects on growth performance, gut morphology, and microbiota of weaned pigs fed with highly or less digestible diets. J Anim Sci. 2018;96:3302-18.

10. Luo ZF, Fang XL, Shu G, Wang SB, Zhu XT, Gao P, et al. Sorbic acid improves growth performance and regulates insulin-like growth factor system gene expression in swine. J Anim Sci. 2011;89:2356-64.

11. Mair C, Plitzner C, Domig KJ, Schedle K, Windisch W. Impact of inulin and a multispecies probiotic formulation on performance, microbial ecology and concomitant fermentation patterns in newly weaned piglets. J Anim Physiol Anim Nutr (Berl). 2010;94:e164-77.

12. Omogbenigun FO, Nyachoti CM, Slominski BA. The effect of supplementing microbial phytase and organic acids to a corn-soybean based diet fed to early-weaned pigs. J Anim Sci. 2003;81:1806-13.

13. Upadhaya SD, Park JW, Lee JH, Kim IH. Efficacy of beta-mannanase supplementation to corn-soya bean meal-based diets on growth performance, nutrient digestibility, blood urea nitrogen, faecal coliform and lactic acid bacteria and faecal noxious gas emission in growing pigs. Arch Anim Nutr. 2016;70:33-43.

14. Walsh MC, Sholly DM, Hinson RB, Saddoris KL, Sutton AL, Radcliffe JS, et al. Effects of water and diet acidification with and without antibiotics on weanling pig growth and microbial shedding. J Anim Sci. 2007;85:1799-808.

15. Yang C, Zhang L, Cao G, Feng J, Yue M, Xu Y, et al. Effects of dietary supplementation with essential oils and organic acids on the growth performance, immune system, fecal volatile fatty acids, and microflora community in weaned piglets. J Anim Sci. 2019;97:133-43.

16. Yen HC, Lai WK, Lin CS, Chiang SH. Medium-chain triglyceride as an alternative of in-feed colistin sulfate to improve growth performance and intestinal microbial environment in newly weaned pigs. Anim Sci J. 2015;86:99-104.

17. Espinosa CD, Fry RS, Usry JL, Stein HH. Copper hydroxychloride improves growth performance and reduces diarrhea frequency of weanling pigs fed a corn-soybean meal diet but does not change apparent total tract digestibility of energy and acid hydrolyzed ether extract. J Anim Sci. 2017;95:5447-54.

18. Han XY, Ma YF, Lv MY, Wu ZP, Qian LC. Chitosan-zinc chelate improves intestinal structure and mucosal function and decreases apoptosis in ileal mucosal epithelial cells in weaned pigs. Br J Nutr. 2014;111:1405-11.

19. Jlali M, Briens M, Rouffineau F, Geraert PA, Mercier Y. Evaluation of the efficacy of 2-hydroxy-4-methylselenobutanoic acid on growth performance and tissue selenium retention in growing pigs. J Anim Sci. 2014;92:182-8.

20. Li MZ, Huang JT, Tsai YH, Mao SY, Fu CM, Lien TF. Nanosize of zinc oxide and the effects on zinc digestibility, growth performances, immune response and serum parameters of weanling piglets. Anim Sci J. 2016;87:1379-85.

21. Marcolla CS, Holanda DM, Ferreira SV, Rocha GC, Serao NVL, Duarte MS, et al. Chromium, CLA, and ractopamine for finishing pigs. J Anim Sci. 2017;95:4472-80.

22. Mateo RD, Spallholz JE, Elder R, Yoon I, Kim SW. Efficacy of dietary selenium sources on growth and carcass characteristics of growing-finishing pigs fed diets containing high endogenous selenium. J Anim Sci. 2007;85:1177-83.

23. Choi JY, Kim JS, Ingale SL, Kim KH, Shinde PL, Kwon IK, et al. Effect of potential multimicrobe probiotic product processed by high drying temperature and antibiotic on performance of weanling pigs. J Anim Sci. 2011;89:1795-804.

24. Chu GM, Lee SJ, Jeong HS, Lee SS. Efficacy of probiotics from anaerobic microflora with prebiotics on growth performance and noxious gas emission in growing pigs. Anim Sci J. 2011;82:282-90.

25. Hancox LR, Le Bon M, Richards PJ, Guillou D, Dodd CE, Mellits KH. Effect of a single dose of *Saccharomyces cerevisiae* var. boulardii on the occurrence of porcine neonatal diarrhoea. Animal. 2015;9:1756-9.

26. Kim J, Kim J, Kim Y, Oh S, Song M, Choe JH, et al. Influences of quorum-quenching probiotic bacteria on the gut microbial community and immune function in weaning pigs. Anim Sci J. 2018;89:412-22.

27. Lan RX, Lee SI, Kim IH. Effects of multistrain probiotics on growth performance, nutrient digestibility, blood profiles, faecal microbial shedding, faecal score and noxious gas emission in weaning pigs. J Anim Physiol Anim Nutr (Berl). 2016;100:1130-8.

28. Li J, Kim IH. Effects of *Saccharomyces cerevisiae* cell wall extract and poplar propolis ethanol extract supplementation on growth performance, digestibility, blood profile, fecal microbiota and fecal noxious gas emissions in growing pigs. Anim Sci J. 2014;85:698-705.

29. Li J, Li D, Gong L, Ma Y, He Y, Zhai H. Effects of live yeast on the performance, nutrient digestibility, gastrointestinal microbiota and concentration of volatile fatty acids in weanling pigs. Arch Anim Nutr. 2006;60:277-88.

30. Liu W, Devi S, Park J, Kim I. Effects of complex probiotic supplementation in growing pig diets with and without palm kernel expellers on growth performance, nutrient digestibility, blood parameters, fecal microbial shedding and noxious gas emission. Anim Sci J. 2018;89:552-60.

31. Shen YB, Piao XS, Kim SW, Wang L, Liu P, Yoon I, et al. Effects of yeast culture supplementation on growth performance, intestinal health, and immune response of nursery pigs. J Anim Sci. 2009;87:2614-24.

32. van Heugten E, Funderburke DW, Dorton KL. Growth performance, nutrient digestibility, and fecal microflora in weanling pigs fed live yeast. J Anim Sci. 2003;81:1004-12.

33. Hu S, Wang Y, Wen X, Wang L, Jiang Z, Zheng C. Effects of low-molecular-weight chitosan on the growth performance, intestinal morphology, barrier function, cytokine expression and antioxidant system of weaned piglets. BMC Vet Res. 2018;14:215.

34. Manzanilla EG, Nofrarias M, Anguita M, Castillo M, Perez JF, Martin-Orue SM, et al. Effects of butyrate, avilamycin, and a plant extract combination on the intestinal equilibrium of early-weaned pigs. J Anim Sci. 2006;84:2743-51.

35. Pan B, Li D, Piao X, Zhang L, Guo L. Effect of dietary supplementation with alpha-galactosidase preparation and stachyose on growth performance, nutrient digestibility and intestinal bacterial populations of piglets. Arch Tierernahr. 2002;56:327-37.

36. Partanen K, Siljander-Rasi H, Pentikainen J, Pelkonen S, Fossi M. Effects of weaning age and formic acid-based feed additives on pigs from weaning to slaughter. Arch Anim Nutr. 2007;61:336-56.

37. Qian L, Yue X, Hu L, Ma Y, Han X. Changes in diarrhea, nutrients apparent digestibility, digestive enzyme activities of weaned piglets in response to chitosan-zinc chelate. Anim Sci J. 2016;87:564-9.

38. van der Peet-Schwering CM, Jansman AJ, Smidt H, Yoon I. Effects of yeast culture on performance, gut integrity, and blood cell composition of weanling pigs. J Anim Sci. 2007;85:3099-109.

39. Van Nevel CJ, Decuypere JA, Dierick NA, Molly K. Incorporation of galactomannans in the diet of newly weaned piglets: effect on bacteriological and some morphological characteristics of the small intestine. Arch Anim Nutr. 2005;59:123-38.

40. Walsh AM, Sweeney T, Bahar B, Flynn B, O'Doherty JV. The effects of supplementing varying molecular weights of chitooligosaccharide on performance, selected microbial populations and nutrient digestibility in the weaned pig. Animal. 2013;7:571-9.

41. Yeh HS, Weng BC, Lien TF. Effects of Chinese traditional herbal medicine complex supplementation on the growth performance, immunity and serum traits of pigs. Anim Sci J. 2011;82:747-52.

42. Zhao P, Piao X, Zeng Z, Li P, Xu X, Wang H. Effect of *Forsythia suspensa* extract and chito-oligosaccharide alone or in combination on performance, intestinal barrier function, antioxidant capacity and immune characteristics of weaned piglets. Anim Sci J. 2017;88:854-62.

43. Lee DN, Liu SR, Chen YT, Wang RC, Lin SY, Weng CF. Effects of diets supplemented with organic acids and nucleotides on growth, immune responses and digestive tract development in weaned pigs. J Anim Physiol Anim Nutr (Berl). 2007;91:508-18.

44. Superchi P, Saleri R, Borghetti P, De Angelis E, Ferrari L, Cavalli V, et al. Effects of dietary nucleotide supplementation on growth performance and hormonal and immune responses of piglets. Animal. 2012;6:902-8.

45. Waititu SM, Yin F, Patterson R, Yitbarek A, Rodriguez-Lecompte JC, Nyachoti CM. Dietary supplementation with a nucleotide-rich yeast extract modulates gut immune response and microflora in weaned pigs in response to a sanitary challenge. Animal. 2017;11:2156-64.

46. Wang Y, Shan T, Xu Z, Liu J, Feng J. Effect of lactoferrin on the growth performance, intestinal morphology, and expression of PR-39 and protegrin-1 genes in weaned piglets. J Anim Sci. 2006;84:2636-41.

47. Yi H, Wang L, Xiong Y, Wen X, Wang Z, Yang X, et al. Effects of *Lactobacillus reuteri* LR1 on the growth performance, intestinal morphology, and intestinal barrier function in weaned pigs. J Anim Sci. 2018;96:2342-51.

48. Chen J, Li Y, Yu B, Chen D, Mao X, Zheng P, et al. Dietary chlorogenic acid improves growth performance of weaned pigs through maintaining antioxidant capacity and intestinal digestion and absorption function. J Anim Sci. 2018;96:1108-18.

49. Clarke LC, Sweeney T, Curley E, Duffy SK, Rajauria G, O'Doherty JV. The variation in chemical composition of barley feed with or without enzyme supplementation influences nutrient digestibility and subsequently affects performance in piglets. J Anim Physiol Anim Nutr (Berl). 2018;102:799-809.

50. Davis ME, Maxwell CV, Erf GF, Brown DC, Wistuba TJ. Dietary supplementation with phosphorylated mannans improves growth response and modulates immune function of weanling pigs. J Anim Sci. 2004;82:1882-91.

51. Fang ZF, Peng J, Liu ZL, Liu YG. Responses of non-starch polysaccharide-degrading enzymes on digestibility and performance of growing pigs fed a diet based on corn, soya bean meal and Chinese double-low rapeseed meal. J Anim Physiol Anim Nutr (Berl). 2007;91:361-8.

52. Jo JK, Ingale SL, Kim JS, Kim YW, Kim KH, Lohakare JD, et al. Effects of exogenous enzyme supplementation to corn- and soybean meal-based or complex diets on growth performance, nutrient digestibility, and blood metabolites in growing pigs. J Anim Sci. 2012;90:3041-8.

53. Lan R, Li T, Kim I. Effects of xylanase supplementation on growth performance, nutrient digestibility, blood parameters, fecal microbiota, fecal score and fecal noxious gas emission of weaning pigs fed corn-soybean meal-based diet. Anim Sci J. 2017;88:1398-405.

54. Molist F, Hermes RG, de Segura AG, Martin-Orue SM, Gasa J, Manzanilla EG, et al. Effect and interaction between wheat bran and zinc oxide on productive performance and intestinal health in post-weaning piglets. Br J Nutr. 2011;105:1592-600.

55. Omogbenigun FO, Nyachoti CM, Slominski BA. Dietary supplementation with multienzyme preparations improves nutrient utilization and growth performance in weaned pigs. J Anim Sci. 2004;82:1053-61.

56. Owusu-Asiedu A, Kiarie E, Peron A, Woyengo TA, Simmins PH, Nyachoti CM. Growth performance and nutrient digestibilities in nursery pigs receiving varying doses of xylanase and beta-glucanase blend in pelleted wheat- and barley-based diets. J Anim Sci. 2012;90 Suppl 4:92-4.

57. Shan T, Wang Y, Wang Y, Liu J, Xu Z. Effect of dietary lactoferrin on the immune functions and serum iron level of weanling piglets. J Anim Sci. 2007;85:2140-6.

58. Taylor AE, Bedford MR, Miller HM. The effects of xylanase on grower pig performance, concentrations of volatile fatty acids and peptide YY in portal and peripheral blood. Animal. 2018;12:2499-504.

59. Vahjen W, Osswald T, Schafer K, Simon O. Comparison of a xylanase and a complex of non starch polysaccharide-degrading enzymes with regard to performance and bacterial metabolism in weaned piglets. Arch Anim Nutr. 2007;61:90-102.

60. Walk CL, Srinongkote S, Wilcock P. Influence of a microbial phytase and zinc oxide on young pig growth performance and serum minerals. J Anim Sci. 2013;91:286-91.

61. Zhang GG, Yang ZB, Wang Y, Yang WR, Zhou HJ. Effects of dietary supplementation of multi-enzyme on growth performance, nutrient digestibility, small intestinal digestive enzyme activities, and large intestinal selected microbiota in weanling pigs. J Anim Sci. 2014;92:2063-9.

62. Zhang S, Song J, Deng Z, Cheng L, Tian M, Guan W. Effects of combined alpha-galactosidase and xylanase supplementation on nutrient digestibility and growth performance in growing pigs. Arch Anim Nutr. 2017;71:441-54.

63. Long Y, Lin S, Zhu J, Pang X, Fang Z, Lin Y, et al. Effects of dietary lysozyme levels on growth performance, intestinal morphology, non-specific immunity and mRNA expression in weanling piglets. Anim Sci J. 2016;87:411-8.

64. Oliver WT, Wells JE. Lysozyme as an alternative to antibiotics improves growth performance and small intestinal morphology in nursery pigs. J Anim Sci. 2013;91:3129-36.

65. Oliver WT, Wells JE, Maxwell CV. Lysozyme as an alternative to antibiotics improves performance in nursery pigs during an indirect immune challenge. J Anim Sci. 2014;92:4927-34.

66. Hosseindoust AR, Lee SH, Kim JS, Choi YH, Kwon IK, Chae BJ. Productive performance of weanling piglets was improved by administration of a mixture of bacteriophages, targeted to control coliforms and *Clostridium* spp. shedding in a challenging environment. J Anim Physiol Anim Nutr (Berl). 2017;101:e98-107.

67. Kim JS, Hosseindoust A, Lee SH, Choi YH, Kim MJ, Lee JH, et al. Bacteriophage cocktail and multi-strain probiotics in the feed for weanling pigs: effects on intestine morphology and targeted intestinal coliforms and *Clostridium*. Animal. 2017;11:45-53.

68. Begum M, Hossain MM, Kim IH. Effects of fenugreek seed extract supplementation on growth performance, nutrient digestibility, diarrhoea scores, blood profiles, faecal microflora and faecal noxious gas emission in weanling piglets. J Anim Physiol Anim Nutr (Berl). 2016;100:1121-9.

69. Chen J, Kang B, Zhao Y, Yao K, Fu C. Effects of natural dietary supplementation with *Macleaya cordata* extract containing sanguinarine on growth performance and gut health of early-weaned piglets. J Anim Physiol Anim Nutr (Berl). 2018;102:1666-74.

70. Cheng C, Xia M, Zhang X, Wang C, Jiang S, Peng J. Supplementing oregano essential oil in a reduced-protein diet improves growth performance and nutrient digestibility by modulating intestinal bacteria, intestinal morphology, and antioxidative capacity of growing-finishing pigs. Animals (Basel). 2018;8:159.

71. Di Giancamillo A, Rossi R, Pastorelli G, Deponti D, Carollo V, Casamassima D, et al. The effects of dietary verbascoside on blood and liver oxidative stress status induced by a high n-6 polyunsaturated fatty acids diet in piglets. J Anim Sci. 2015;93:2849-59.

72. Ilsley SE, Miller HM, Kamel C. Effects of dietary quillaja saponin and curcumin on the performance and immune status of weaned piglets. J Anim Sci. 2005;83:82-8.

73. Jeong JS, Kim IH. Effect of probiotic bacteria-fermented medicinal plants (*Gynura procumbens*, *Rehmannia glutinosa*, *Scutellaria baicalensis*) as performance enhancers in growing pigs. Anim Sci J. 2015;86:603-9.

74. Kang SN, Chu GM, Song YM, Jin SK, Hwang IH, Kim IS. The effects of replacement of antibiotics with by-products of oriental medicinal plants on growth performance and meat qualities in fattening pigs. Anim Sci J. 2012;83:245-51.

75. Lei XJ, Lee SI, Kim IH. Effects of different levels of dietary protein with or without plant extract YGF251 on growth performance, nutrient digestibility, blood profiles, fecal microbial shedding, and fecal gas emission in growing pigs. Anim Sci J. 2019;90:547-53.

76. Manzanilla EG, Perez JF, Martin M, Kamel C, Baucells F, Gasa J. Effect of plant extracts and formic acid on the intestinal equilibrium of early-weaned pigs. J Anim Sci. 2004;82:3210-8.

77. Nowak P, Kasprowicz-Potocka M, Zaworska A, Nowak W, Stefanska B, Sip A, et al. The effect of eubiotic feed additives on the performance of growing pigs and the activity of intestinal microflora. Arch Anim Nutr. 2017;71:455-69.

78. Schone F, Vetter A, Hartung H, Bergmann H, Biertumpfel A, Richter G, et al. Effects of essential oils from fennel (*Foeniculi aetheroleum*) and caraway (*Carvi aetheroleum*) in pigs. J Anim Physiol Anim Nutr (Berl). 2006;90:500-10.

79. Wang DF, Zhou LL, Zhou HL, Hou GY, Zhou X, Li W. Effects of *Piper sarmentosum* extract on the growth performance, antioxidant capability and immune response in weaned piglets. J Anim Physiol Anim Nutr (Berl). 2017;101:105-12.

80. Yan L, Kim IH. Effects of dietary supplementation of fermented garlic powder on growth performance, apparent total tract digestibility, blood characteristics and faecal microbial concentration in weanling pigs. J Anim Physiol Anim Nutr (Berl). 2013;97:457-64.

81. Yan L, Meng QW, Kim IH. Effects of fermented garlic powder supplementation on growth performance, nutrient digestibility, blood characteristics and meat quality in growing-finishing pigs. Anim Sci J. 2012;83:411-7.

82. Yin FG, Liu YL, Yin YL, Kong XF, Huang RL, Li TJ, et al. Dietary supplementation with *Astragalus* polysaccharide enhances ileal digestibilities and serum concentrations of amino acids in early weaned piglets. Amino Acids. 2009;37:263-70.

83. Cutler SA, Lonergan SM, Cornick N, Johnson AK, Stahl CH. Dietary inclusion of colicin e1 is effective in preventing postweaning diarrhea caused by F18-positive *Escherichia coli* in pigs. Antimicrob Agents Chemother. 2007;51:3830-5.

84. Tang Z, Yin Y, Zhang Y, Huang R, Sun Z, Li T, et al. Effects of dietary supplementation with an expressed fusion peptide bovine lactoferricin-lactoferrampin on performance, immune function and intestinal mucosal morphology in piglets weaned at age 21 d. Br J Nutr. 2009;101:998-1005.

85. Wu S, Zhang F, Huang Z, Liu H, Xie C, Zhang J, et al. Effects of the antimicrobial peptide cecropin AD on performance and intestinal health in weaned piglets challenged with *Escherichia coli*. Peptides. 2012;35:225-30.

86. Xiao H, Tan BE, Wu MM, Yin YL, Li TJ, Yuan DX, et al. Effects of composite antimicrobial peptides in weanling piglets challenged with deoxynivalenol: II. Intestinal morphology and function. J Anim Sci. 2013;91:4750-6.

87. Xiao H, Wu MM, Tan BE, Yin YL, Li TJ, Xiao DF, et al. Effects of composite antimicrobial peptides in weanling piglets challenged with deoxynivalenol: I. Growth performance, immune function, and antioxidation capacity. J Anim Sci. 2013;91:4772-80.

88. Yoon JH, Ingale SL, Kim JS, Kim KH, Lee SH, Park YK, et al. Effects of dietary supplementation of antimicrobial peptide-A3 on growth performance, nutrient digestibility, intestinal and fecal microflora and intestinal morphology in weanling pigs. Anim Feed Sci Technol. 2012;177:98-107.

89. Yoon JH, Ingale SL, Kim JS, Kim KH, Lee SH, Park YK, et al. Effects of dietary supplementation of synthetic antimicrobial peptide-A3 and P5 on growth performance, apparent total tract digestibility of nutrients, fecal and intestinal microflora and intestinal morphology in weanling pigs. J Sci Food Agric. 2013;93:587-92.

90. Zanetti M, Storici P, Tossi A, Scocchi M, Gennaro R. Molecular cloning and chemical synthesis of a novel antibacterial peptide derived from pig myeloid cells. J Biol Chem. 1994;269:7855-8.

91. Tamamura H, Murakami T, Horiuchi S, Sugihara K, Otaka A, Takada W, et al. Synthesis of protegrin-related peptides and their antibacterial and anti-human immunodeficiency virus activity. Chem Pharm Bull (Tokyo). 1995;43:853-8.

92. Tossi A, Scocchi M, Zanetti M, Storici P, Gennaro R. PMAP-37, a novel antibacterial peptide from pig myeloid cells. cDNA cloning, chemical synthesis and activity. Eur J Biochem. 1995;228:941-6.

93. Lee JY, Boman A, Sun CX, Andersson M, Jornvall H, Mutt V, et al. Antibacterial peptides from pig intestine: isolation of a mammalian cecropin. Proc Natl Acad Sci U S A. 1989;86:9159-62.

94. Kim JB, Halverson T, Basir YJ, Dulka J, Knoop FC, Abel PW, et al. Purification and characterization of antimicrobial and vasorelaxant peptides from skin extracts and skin secretions of the North American pig frog *Rana grylio*. Regul Pept. 2000;90:53-60.

95. Jin F, Sun Q, Xu X, Li L, Gao G, Xu Y, et al. cDNA cloning and characterization of the antibacterial peptide cecropin 1 from the diamondback moth, *Plutella xylostella* L. Protein Expr Purif. 2012;85:230-8.

96. Xia L, Liu Z, Ma J, Sun S, Yang J, Zhang F. Expression, purification and characterization of cecropin antibacterial peptide from *Bombyx mori* in *Saccharomyces cerevisiae*. Protein Expr Purif. 2013;90:47-54.

97. Wang H, Meng XL, Xu JP, Wang J, Wang H, Ma CW. Production, purification, and characterization of the cecropin from *Plutella xylostella*, pxCECA1, using an intein-induced self-cleavable system in *Escherichia coli*. Appl Microbiol Biotechnol. 2012;94:1031-9.

98. Hao X, Yang H, Wei L, Yang S, Zhu W, Ma D, et al. Amphibian cathelicidin fills the evolutionary gap of cathelicidin in vertebrate. Amino Acids. 2012;43:677-85.

99. Yu H, Cai S, Gao J, Zhang S, Lu Y, Qiao X, et al. Identification and polymorphism discovery of the cathelicidins, Lf-CATHs in ranid amphibian (*Limnonectes fragilis*). FEBS J. 2013;280:6022-32.

100. Wei L, Yang J, He X, Mo G, Hong J, Yan X, et al. Structure and function of a potent lipopolysaccharide-binding antimicrobial and anti-inflammatory peptide. J Med Chem. 2013;56:3546-56.

101. Unlu M, Ergene E, Unlu GV, Zeytinoglu HS, Vural N. Composition, antimicrobial activity and *in vitro* cytotoxicity of essential oil from *Cinnamomum zeylanicum* Blume (*Lauraceae*). Food Chem Toxicol. 2010;48:3274-80.

102. Gazim ZC, Amorim AC, Hovell AM, Rezende CM, Nascimento IA, Ferreira GA, et al. Seasonal variation, chemical composition, and analgesic and antimicrobial activities of the essential oil from leaves of *Tetradenia riparia* (*Hochst.*) Codd in Southern Brazil. Molecules. 2010;15:5509-24.

103. Mushi NF, Mbwambo ZH, Innocent E, Tewtrakul S. Antibacterial, anti-HIV-1 protease and cytotoxic activities of aqueous ethanolic extracts from *Combretum adenogonium* Steud. Ex A. Rich (*Combretaceae*). BMC Complement Altern Med. 2012;12:163.

104. Fabri RL, Coimbra ES, Almeida AC, Siqueira EP, Alves TM, Zani CL, et al. Essential oil of *Mitracarpus frigidus* as a potent source of bioactive compounds. An Acad Bras Cienc. 2012;84:1073-80.

105. Mathlouthi N, Bouzaienne T, Oueslati I, Recoquillay F, Hamdi M, Urdaci M, et al. Use of rosemary, oregano, and a commercial blend of essential oils in broiler chickens: *in vitro* antimicrobial activities and effects on growth performance. J Anim Sci. 2012;90:813-23.

106. Taleb MH, Abdeltawab NF, Shamma RN, Abdelgayed SS, Mohamed SS, Farag MA, et al. *Origanum vulgare* L. essential oil as a potential anti-acne topical nanoemulsion-*in vitro* and *in vivo* study. Molecules. 2018;23:2164.

107. Ade SC, da Silva MV, Gomes FS, Paiva PM, Malafaia CB, da Silva TD, et al. Purification, characterization and antibacterial potential of a lectin isolated from *Apuleia leiocarpa* seeds. Int J Biol Macromol. 2015;75:402-8.

108. Rivero-Cruz I, Duarte G, Navarrete A, Bye R, Linares E, Mata R. Chemical composition and antimicrobial and spasmolytic properties of *Poliomintha longiflora* and *Lippia graveolens* essential oils. J Food Sci. 2011;76:C309-17.

109. Falcone PM, Mastromatteo M, Del Nobile MA, Corbo MR, Sinigaglia M. Evaluating *in vitro* antimicrobial activity of thymol toward hygiene-indicating and pathogenic bacteria. J Food Prot. 2007;70:425-31.

110. Baskaran SA, Kazmer GW, Hinckley L, Andrew SM, Venkitanarayanan K. Antibacterial effect of plant-derived antimicrobials on major bacterial mastitis pathogens *in vitro*. J Dairy Sci. 2009;92:1423-9.

111. Tayel AA, El-Tras WF, Moussa SH, El-Sabbagh SM. Surface decontamination and quality enhancement in meat steaks using plant extracts as natural biopreservatives. Foodborne Pathog Dis. 2012;9:755-61.

112. Boskovic M, Djordjevic J, Ivanovic J, Janjic J, Zdravkovic N, Glisic M, et al. Inhibition of salmonella by thyme essential oil and its effect on microbiological and sensory properties of minced pork meat packaged under vacuum and modified atmosphere. Int J Food Microbiol. 2017;258:58-67.

113. Chen H, Zhong Q. Lactobionic acid enhances the synergistic effect of nisin and thymol against *Listeria monocytogenes* Scott A in tryptic soy broth and milk. Int J Food Microbiol. 2017;260:36-41.

114. Bukvicki D, Stojkovic D, Sokovic M, Vannini L, Montanari C, Pejin B, et al. *Satureja horvatii* essential oil: *in vitro* antimicrobial and antiradical properties and *in situ* control of *Listeria monocytogenes* in pork meat. Meat Sci. 2014;96:1355-60.

115. He L, Zou L, Yang Q, Xia J, Zhou K, Zhu Y, et al. Antimicrobial activities of nisin, tea polyphenols, and chitosan and their combinations in chilled mutton. J Food Sci. 2016;81:M1466-71.

116. Bahiense JB, Marques FM, Figueira MM, Vargas TS, Kondratyuk TP, Endringer DC, et al. Potential anti-inflammatory, antioxidant and antimicrobial activities of *Sambucus australis*. Pharm Biol. 2017;55:991-7.

117. Ghasemzadeh A, Jaafar HZ, Rahmat A, Ashkani S. Secondary metabolites constituents and antioxidant, anticancer and antibacterial activities of *Etlingera elatior* (Jack) R.M.Sm grown in different locations of Malaysia. BMC Complement Altern Med. 2015;15:335.

118. Ma Q, Davidson PM, Zhong Q. Antimicrobial properties of lauric arginate alone or in combination with essential oils in tryptic soy broth and 2% reduced fat milk. Int J Food Microbiol. 2013;166:77-84.

119. de Araujo AA, Soares LA, Ferreira MR, Neto MA, da Silva GR, de Araujo RF, Jr., et al. Quantification of polyphenols and evaluation of antimicrobial, analgesic and anti-inflammatory activities of aqueous and acetone-water extracts of *Libidibia ferrea*, *Parapiptadenia rigida* and *Psidium guajava*. J Ethnopharmacol. 2014;156:88-96.

120. Brahmachari G, Mandal NC, Roy R, Ghosh R, Barman S, Sarkar S, et al. A new pentacyclic triterpene with potent antibacterial activity from *Limnophila indica* Linn. (Druce). Fitoterapia. 2013;90:104-11.

121. Alonso-Castro AJ, Gonzalez-Chavez MM, Zapata-Morales JR, Verdinez-Portales AK, Sanchez-Recillas A, Ortiz-Andrade R, et al. Antinociceptive activity of ent-dihydrotucumanoic acid isolated from *Gymnosperma glutinosum* spreng less. Drug Dev Res. 2017;78:340-8.

122. Kelsey JA, Bayles KW, Shafii B, McGuire MA. Fatty acids and monoacylglycerols inhibit growth of *Staphylococcus aureus*. Lipids. 2006;41:951-61.

123. Burt SA, Adolfse SJ, Ahad DS, Tersteeg-Zijderveld MH, Jongerius-Gortemaker BG, Post JA, et al. Cinnamaldehyde, carvacrol and organic acids affect gene expression of selected oxidative stress and inflammation markers in IPEC-J2 cells exposed to *Salmonella typhimurium*. Phytother Res. 2016;30:1988-2000.

124. Shaalan MI, El-Mahdy MM, Theiner S, El-Matbouli M, Saleh M. *In vitro* assessment of the antimicrobial activity of silver and zinc oxide nanoparticles against fish pathogens. Acta Vet Scand. 2017;59:49.

125. Chun W, Hancock RE. Action of lysozyme and nisin mixtures against lactic acid bacteria. Int J Food Microbiol. 2000;60:25-32.

126. Wang Q, Zhang L, Zhao J, You L, Wu H. Two goose-type lysozymes in *Mytilus galloprovincialis*: possible function diversification and adaptive evolution. PLoS One. 2012;7:e45148.
